# Risk factors for respiratory syncytial virus associated with acute lower respiratory infection in children under five years: Systematic review and meta–analysis

**DOI:** 10.7189/jogh.05.020416

**Published:** 2015-12

**Authors:** Ting Shi, Evelyn Balsells, Elizabeth Wastnedge, Rosalyn Singleton, Zeba A Rasmussen, Heather J Zar, Barbara A Rath, Shabir A Madhi, Stuart Campbell, Linda Cheyenne Vaccari, Lisa R Bulkow, Elizabeth D Thomas, Whitney Barnett, Christian Hoppe, Harry Campbell, Harish Nair

**Affiliations:** 1 Centre for Global Health Research, Usher Institute of Population Health Sciences and Informatics, University of Edinburgh, Edinburgh, Scotland, United Kingdom; 2 Arctic Investigations Program, Division of Preparedness and Emerging Infectious, National Centre for Emerging and Zoonotic Infectious Diseases (NCEZID), Centres for Disease Control and Prevention (CDC), Anchorage, AK, USA; 3 Alaska Native Tribal Health Consortium, Anchorage, AK, USA; 4 Fogarty International Center, National Institutes of Health, Bethesda MD, USA; 5 Department of Paediatrics and Child Health, Red Cross War Memorial Children’s Hospital and MRC Unit on Child & Adolescent Health, University of Cape Town, South Africa; 6 Department of Pediatrics, Charité University Medical Center, Berlin, Germany; 7 Medical Research Council: Respiratory and Meningeal Pathogens Research Unit, University of the Witwatersrand, Johannesburg, South Africa; 8 Department of Science and Technology/National Research Foundation: Vaccine Preventable Diseases, University of the Witwatersrand, Johannesburg, South Africa; 9 Centre for Respiratory Diseases and Meningitis, National Institute for Communicable Diseases of the National Health Laboratory Service, Johannesburg, South Africa; 10 Centre for Population Health Sciences, Usher Institute of Population Health Sciences and Informatics, University of Edinburgh, Edinburgh, Scotland, United Kingdom; 11 Centre for Medical Informatics, Usher Institute of Population Health Sciences and Informatics, University of Edinburgh, Edinburgh, Scotland, United Kingdom; 12 Public Health Foundation of India, New Delhi, India; * Joint last authorship

## Abstract

**Background:**

Respiratory syncytial virus (RSV) is the most common pathogen identified in young children with acute lower respiratory infection (ALRI) as well as an important cause of hospital admission. The high incidence of RSV infection and its potential severe outcome make it important to identify and prioritise children who are at higher risk of developing RSV–associated ALRI. We aimed to identify risk factors for RSV–associated ALRI in young children.

**Methods:**

We carried out a systematic literature review across 4 databases and obtained unpublished studies from RSV Global Epidemiology Network (RSV GEN) collaborators. Quality of all eligible studies was assessed according to modified GRADE criteria. We conducted meta–analyses to estimate odds ratios with 95% confidence intervals (CI) for individual risk factors.

**Results:**

We identified 20 studies (3 were unpublished data) with “good quality” that investigated 18 risk factors for RSV–associated ALRI in children younger than five years old. Among them, 8 risk factors were significantly associated with RSV–associated ALRI. The meta–estimates of their odds ratio (ORs) with corresponding 95% confidence intervals (CI) are prematurity 1.96 (95% CI 1.44–2.67), low birth weight 1.91 (95% CI 1.45–2.53), being male 1.23 (95% CI 1.13–1.33), having siblings 1.60 (95% CI 1.32–1.95), maternal smoking 1.36 (95% CI 1.24–1.50), history of atopy 1.47 (95% CI 1.16–1.87), no breastfeeding 2.24 (95% CI 1.56–3.20) and crowding 1.94 (95% CI 1.29–2.93). Although there were insufficient studies available to generate a meta–estimate for HIV, all articles (irrespective of quality scores) reported significant associations between HIV and RSV–associated ALRI.

**Conclusions:**

This study presents a comprehensive report of the strength of association between various socio–demographic risk factors and RSV–associated ALRI in young children. Some of these amenable risk factors are similar to those that have been identified for (all cause) ALRI and thus, in addition to the future impact of novel RSV vaccines, national action against ALRI risk factors as part of national control programmes can be expected to reduce burden of disease from RSV. Further research which identifies, accesses and analyses additional unpublished RSV data sets could further improve the precision of these estimates.

Acute lower respiratory infection (ALRI), including pneumonia and bronchiolitis, remains the leading cause of childhood hospitalisation and mortality [1], primarily within developing countries [2]. It is estimated that in 2010, there were about 120.4 million episodes of ALRI and about 14.1 million respective episodes of severe ALRI in children younger than 5 years [3]. It is also estimated that there were 1.4 million pneumonia deaths in this age group that year (which decreased to 936 000 in 2013) [4].

Globally, respiratory syncytial virus (RSV) is the most common pathogen identified in young children with ALRI, as well as an important cause of hospital admissions [5]. It is estimated that in 2005 there were about 33.8 million new episodes of ALRI which were RSV positive in children younger than 5 years and about 10% of these were severe enough to warrant hospitalisation. It is also estimated that RSV attributable mortality in children younger than 5 years was around 53 255 in–hospital deaths and up to 199 260 overall deaths globally in 2005, with 99% of these occurring in developing countries.

RSV is known to be more likely to have a severe outcome in children with certain pre–existing chronic medical conditions, resulting in higher rate of hospitalisation and higher risk of death. A case-control study in southwest Alaska indicated that underlying medical conditions, such as prematurity, chronic lung disease and heart disease, were associated with an increased risk of RSV hospitalisation [6]. Another systematic review reported that the case fatality ratio among children hospitalised with RSV infection was higher in children with chronic lung disease, congenital heart disease or prematurity, compared to otherwise healthy children [7]. The high incidence of RSV infection, as well as its potentially severe outcome, makes it important to identify and prioritise children at high risk of developing RSV–associated ALRI.

To date, there has been only one systematic review published over a decade ago that assessed the strength of association between various risk factors and RSV–associated ALRI [8]. There have been no recent comprehensive systematic reviews that included the recent literatures reporting the association of various putative risk factors and RSV–associated ALRI in young children. Therefore, we conducted a systematic review to identify studies investigating the association between potential risk factors and RSV–associated ALRI in children younger than five years. We aimed to assess the quality of available evidence and present summary meta–estimates of the strength of association between multiple risk factors and RSV–associated ALRI to identify targeted prevention strategies.

## METHODS

### Search strategy and selection criteria

We conducted a systematic review according to the PRISMA guidelines. The search was conducted across the following electronic databases: Medline, Embase, Global Health and LILACS. The search terms used are detailed in Appendix S1 in **Online Supplementary Document(Online Supplementary Document)**. We further hand searched the reference lists of relevant papers for eligible articles. All searches were limited to between January 1995 and July 2015, and there were no publication status or language restrictions applied. Eligible studies were observational studies or randomized controlled trials that assessed the relationship between RSV–associated ALRI and risk factors of interest. **Table 1** provides the selection criteria in detail.

**Table 1:** Eligibility criteria for selection of studies in the systematic review

**Inclusion criteria:**
**Published from January 1995 to July 2015**
**Providing data for children younger than 5 y**
**Focusing on children with a diagnosis of ALRI and laboratory confirmed RSV illness**
**Reporting association between socio–demographic risk factors and RSV–associated ALRI**
**Sample size ≥50 children below 5 y**
**Study design–observational studies (case–control or cohort) or randomized controlled trials (placebo arm only)**
**Reporting results on risk factors based on univariable or multivariable analysis**
**Exclusion criteria:**
**Definitions used for ALRI or risk factors, not clearly stated or inconsistently applied**
**Focusing on risk factors solely among high–risk study population (eg, preterm babies, children with congenital heart disease, chronic lung disease and immunosuppression etc.)**
**Ineligible control group (eg, RSV negative ALRI cases, children hospitalised for acute infections)**
**Methods for analysis not clearly reported**

ALRI – acute lower respiratory infection, RSV – respiratory syncytial virus

Two investigators (TS and EB) conducted independent literature searches and extracted data using standardised data extraction template. Any discordance or uncertainties regarding relevance or inclusion were arbitrated by HN.

Data from unpublished studies provided by RSV Global Epidemiology Network (RSV GEN) collaborators were reviewed (by TS) for quality and inconsistencies. RSV GEN is a working group established to collect unpublished data in order to evaluate the disease burden of RSV worldwide.

The protocol of this review was published in PROSPERO database (No. CRD42015017923).

### Definitions

We used RSV–associated ALRI as the outcome of interest, which includes clinical pneumonia and bronchiolitis. This was to recognize these common manifestations in young children with viral ALRI [9], and the limitations of the WHO case definition to reliably differentiate bronchiolitis from pneumonia [1]. ALRI was defined as cough or dyspnoea with age–related tachypnoea, while severe ALRI was defined as cough or dyspnoea with lower chest wall indrawing or an acute respiratory infection severe enough to warrant hospital admission. The control group was defined as children without RSV infection (children without respiratory symptoms) or healthy (children without any symptoms). Countries were categorised as developing or industrialised according to the “Levels and trends in child mortality–report 2014” by UNICEF [10]. The Alaskan native population in America was considered to share some epidemiological features to populations in developing countries with similar socioeconomic and demographic risk factors for respiratory infections in both populations [11].

We recognized that the definitions for some risk factors used in the included studies varied substantially (Appendix S2 in **Online Supplementary Document(Online Supplementary Document)**). Where there were several slightly different definitions (which may result in differing strengths of association between risk factor and outcome), we pooled the studies into one meta–analysis (where possible) and then conducted a sensitivity analysis. The definitions of risk factors included in the following meta–analysis were listed in **Table 2****.**

**Table 2:** List of the various definitions of risk factors for RSV–associated ALRI included in meta–analysis

Risk factor	Definition
**Prematurity:**	Gestational age <37 weeks
	Gestational age <33 weeks
**Low birth weight**	Birth weight <2.5 kg
**Gender**	Male
**Siblings**	Mention of siblings or other children living in the household
**Maternal smoking**	Maternal smoking during pregnancy
**History of atopy**	Positive family history of asthma or atopy
**Low parental education:**	No parent having bachelor’s degree
	Education of primary caregiver: 1–7 y or no schooling
	<12 y maternal education
	<11 y maternal education
**Passive smoking**	Smokers in the household
**Daycare center attendance**	Attendance at daycare center
**Indoor air pollution**	Use of biomass fuels for cooking or a description of indoor smoke
**No breastfeeding**	No breastfeeding
**Crowding**	>7 persons in household
**Multiple births**	Twins or triplets
**HIV**	Confirmed presence of HIV infection in child

ALRI – acute lower respiratory infection, RSV – respiratory syncytial virus, HIV – human immunodeficiency virus, y – years

### Quality assessment

The quality of each study was assessed by using a modified GRADE scoring system [12] focusing on the following aspects: study design, quality of control group, sample size, analysis method, bias, confounding factors and geographical spread of studies (Appendix S3 in **Online Supplementary Document(Online Supplementary Document)**). We calculated the overall score for each study after assessing each criterion as listed above. Studies with cumulative score ≤ lower quartile (25th percentile) of all scores were considered to have “low quality” and they were excluded in the final estimate. Also a sensitivity analysis was run to show whether the results differ when we included these “low–quality” studies.

### Statistical analysis

In included articles or unpublished studies, data about risk summary measure (odds ratio and relative risk) with 95% CI for risk factors of interest were extracted as provided (univariable and multivariable analysis). If such summary data were not reported, we calculated the same (where feasible) using data reported in the paper.

Using STATA (Stata Statistical Software version 11.2, StataCorp LP, College Station TX, USA) we conducted a meta–analysis of risk factor specific odds ratios and reported pooled estimates with corresponding 95% CIs based on random effects model (DerSimonian–Laird method) since significant heterogeneity was found (*I*^2^>80%, *P* < 0.05) [13]. We decided that in the first instance, only results from studies reporting data based on multivariable analysis would be presented. Thereafter, data from studies reporting ORs using univariable analysis were included.

## RESULTS

**Figure 1:**
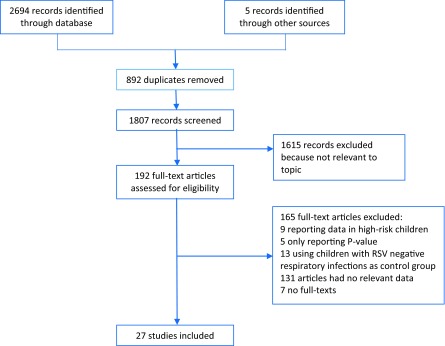
Flow diagram for the selection of studies.

We identified 2694 articles through literature search, of which only 23 studies [6,14-35] fulfilled our strict eligibility criteria. After including an additional 4 studies (Rasmussen, unpublished; Rath, unpublished; Singleton, unpublished; Zar, unpublished) provided by RSV GEN collaborators, 27 studies in total were included in the analysis (**Figure 1**). Six studies provided data on risk factors for RSV–associated ALRI [19,22,27] (Rasmussen, unpublished; Rath, unpublished; Zar, unpublished) and 21 studies provided data for RSV–associated hospitalised ALRI. Fourteen studies were from industrialised countries and 13 studies were from developing countries. A map of locations of these 27 study sites is given in Appendix S5 in **Online Supplementary Document(Online Supplementary Document)**. **Table 3** shows more characteristics of these 27 included studies. According to the modified GRADE scoring system, the scores of included studies varied from 2.5 to 11 with 25th percentile score of 6.25 (Appendix S4 in **Online Supplementary Document(Online Supplementary Document)**). There were 7 studies which had scores ≤6.25 [20,22,31,32,34,35] (Rath, unpublished). **Table 4** presents the final results for risk factors with meta–estimate ORs after excluding “low–quality” studies (20 studies). Forest plots for these risk factors are shown in Appendix S6 in **Online Supplementary Document(Online Supplementary Document)**. Those “low–quality” studies were also included in a sensitivity analysis (Appendix S7 in **Online Supplementary Document(Online Supplementary Document)**).

**Table 3:** Characteristics of 27 included studies

Study	Study period	Study design	Age	Case ascertainment	Case definition	Sample size	RSV detection	Risk factors included
**Hvidovre, Denmark [****29****]**	May 2004–May 2005	Prospective birth cohort	<1y	IP	ARI	217	NPS; PCR	PR, BF, S, PS, MS
**Denmark[****28****]**	1997–2003	Case-control	<18m	IP	ARI	15380	RSV database	M, HOA, DCA, S, MS
**Utrecht, Netherlands [****19****]**	Jan 2006–Dec 2008	Prospective birth cohort	<1y	IP, OP	ALRI	298	Nasal/throat swab; PCR	BF, M, HOA, PE, DCA, MS
**Colorado, USA [****15****]**	1998–2002	Cross-sectional	<4y	IP	P, B	4847	ICD–9 RSV codes	Altitude
**San Marcos, Guatemala [****27****]**	Oct 2002–Dec 2004	Randomized controlled trial	<18m	IP, OP	ARI	NA	NA; IF	IAP
**Kilifi, Kenya [****22****]**	May 2003–Apr 2007	Birth cohort	<4y	C	ALRI	469	NPW; DFA	MB, PE, MA, C, S, PS
**Soweto, South Africa [****20****]**	Mar 1998–Dec 2004	Prospective cohort	<6y	IP	ALRI	39836	NPA; IF	PR, HIV
**South–western Netherlands [****25****]**	Oct 1996–Apr 1999	Retrospective cohort	<2y	IP	ARI	NA	NPA; DFA/culture	PR, LBW, M
**9 perinatal networks, France [****17****]**	Mar 2008 to Apr 2009	Retrospective & prospective cohort	<1y	IP	B	498	NPA; IF	PR
**Kiel, Germany [****31****]**	Jul 1996–Jun 1999	Cross-sectional	<2y	IP	ARI	NA	NPA; PCR	PR
**Bohol, Philippines [****23****]**	Jul 2000–Dec 2004	Retrospective cohort	<5y	IP	ALRI	10913	NA; PCR/culture	M, PE, MA, S
**Townsville, Australia [****24****]**	Jan 1997–Jun 2004	Case-control	<3y	IP	ALRI	750	NPA; DFA	PR, LBW, M, S
**Tennessee, USA [****14****]**	Jul 1989–Jun 1993	Retrospective cohort	<1y	IP	ARI	3553	NA	PR, M, PE, S, MS
**2 Danish counties, Denmark [****21****]**	1990–1994	Case-control	<2y	IP	ALRI	7632	NPA; DFA	PR, LBW, S, MS
**Basque Country, Spain [****16****]**	Jul 1996–Jun 2000	Case-control	<2y	IP	ALRI	14343	NPA; IF	PR, LBW, MB, M,
**Wellington Hospital, New Zealand [****18****]**	June/July–October, 2003–2005	Case-control	<2y	IP	B	11411	NPA; DFA	PR, MB, M, MS
**Alaska, USA [****6****]**	Oct 1993–Sep 1996	Case-control	<3y	IP	ALRI	542	NPA; IF	BF, PE, C, S, S
**3 hospitals in western region, Gambia [****30****]**	1993–1995	Case-control	<5y	IP	ALRI	641	NPA; IF	HOA, M, C, S, PS, M, LPW, IAP
**Italy [****26****]**	Oct–Apr, 2000–2004	Case-control	≤4y	IP	ALRI	437	Nasal sample; IF	PR, LBW, BF, M, HOA, PI, S, PS
**Alaska, USA (Singleton, unpublished)**	Oct 2006–Sep 2007	Case-control	<3y	IP	ALRI	68	NPS; PCR	PR, BF, C, IAP, PS
**Oshikhandass, Pakistan (Rasmussen, unpublished)**	Apr 2012–Mar 2014	Case-control	<5y	C	ALRI	93	NPS; PCR	C, M, PE, S, IAP, PS
**Soweto, South Africa [****32****]**	Mar 1997–Mar 1998	Cross-sectional	2–23m	IP	ALRI	24000	NPA; DFA	HIV
**3 sites, South Africa [****33****]**	Jan 2010–Dec 2011	Cross-sectional	<5y	IP	ALRI	835060	NPA; PCR	HIV
**Alaska, USA [****34****]**	1995–2012	Cross-sectional	<1y	IP	ALRI	NA	NPA; DFA/culture	C, IAP, LPW
**Alaska, USA [****35****]**	2000–2004	Cross-sectional	<1y	IP	ALRI	NA	NPA; DFA/culture	LPW
**Paarl, South Africa (Zar, unpublished)**	Mar 2012–Dec 2014	Prospective cohort	<3y	C	ALRI	159	NPS; RT–PCR	PR, LBW, BF, M, HOA, PE, S, PS, MS, DCA, MA, C, IAP, PI
**Berlin, Germany (Rath, unpublished)**	Apr 2010–Mar 2014	Prospective cohort	<5y	IP, OP	ALRI	666	NPS/NPA; RT–PCR	PR, LBW, M, C

Case ascertainment: IP – inpatient, OP – outpatient; C – community. Case definition: ALRI –acute lower respiratory infection, ARI – acute respiratory infection, P – pneumonia, B – bronchiolitis. RSV detection: NPA – nasopharyngeal aspirate, NPS – nasopharyngeal swab, NPW – nasopharyngeal wash, PCR – polymerase chain reaction, IF – immunofluorescence, DFA – direct fluorescent antibody test, IFA – indirect fluorescent antibody test. Risk factors included: P – prematurity, LBW – low birth weight, BF – no/lack of exclusive breastfeeding, MB – multiple births, M – male, HOA – history of atopy, PE – low parental education, S – siblings, PS – passive smoking, MS – maternal smoking, DCA – daycare center attendance, MA – malnutrition, C – crowding, IAP – indoor air pollution, PI – previous illness, HIV – human immunodeficiency virus, LPW – lack of plumbed water, NA – not available, y – year, m – month

**Table 4:** Meta–estimate of odds ratio for risk factors excluding studies with quality score ≤6.25 (ie, “low–quality”)

Risk factor	Multivariable analysis		Multivariable and univariable analysis	
	No. of studies	Meta–estimate OR (95% confidence interval)	No. of studies	Meta–estimate OR (95% confidence interval)
**Prematurity (gestational age <37 weeks)**	2	–	7	1.96 (1.44–2.67)
**Low birth weight**	2	–	5	1.91 (1.45–2.53)
**Being male**	6	1.32 (1.24–1.40)	12	1.23 (1.13–1.33)
**Siblings**	6	1.53 (1.20–1.95)	11	1.60 (1.32–1.95)
**Maternal smoking**	4	1.34 (1.26–1.42)	7	1.36 (1.24–1.50)
**History of atopy**	1	–	5	1.47 (1.16–1.87)
**Low parental education**	4	1.23 (0.73–2.09)	6	1.40 (0.94–2.08)
**Passive smoking**	4	1.40 (0.65–3.00)	8	1.29 (0.96–1.73)
**Daycare center attendance**	2	–	3	1.61 (0.98–2.64)
**Indoor air pollution**	4	0.69 (0.35–1.37)	5	0.81 (0.42–1.57)
**No breastfeeding**	1	–	3	2.24 (1.56–3.20)
**Crowding (>7 persons in household)**	1	–	3	1.94 (1.29–2.93)

OR – odds ratio

### Prematurity (gestational age <37 weeks)

Prematurity has been defined variously in the included studies. One of the studies [29] used gestational age <38 weeks as definition for prematurity, three studies [14,20,26] used gestational age <36 weeks and nine studies used gestational age <37 weeks. Only studies using definition of gestational age <37 weeks were included in meta–analysis. Among these nine studies, two [16] (Singleton unpublished) reported the associations using multivariable analysis and the others used univariable analysis. Two studies (Singleton, unpublished; Zar, unpublished) were based on settings categorised as developing countries, while the rest were from industrialised countries. One study (Zar, unpublished) was community–based, another (Rath, unpublished) included outpatients and inpatients and the other 7 studies were hospital–based. Two studies [31] (Rath, unpublished) were considered to be “low–quality” studies. After excluding these two studies, the odds ratio meta–estimate was 1.96 (95% CI 1.44–2.67). Alternatively meta–estimate was 1.47 (95% CI 0.98–2.21) if all studies irrespective of quality scores were included.

### Prematurity (gestational age <33 weeks)

This risk factor was considered as a subgroup (more severe) of children with gestational age <37 weeks. Three hospital–based studies [14,21,25] from industrialised countries reported significant associations between prematurity (gestational age <33 weeks) and RSV–associated ALRI using multivariable analysis. The overall odds ratio meta–estimate was 2.68 (95% CI 2.02–3.55). Five additional studies [14,21,25] (Rath unpublished; Zar, unpublished), two of which were from developing countries [20] (Zar, unpublished), reported odds ratios using univariable analysis. The inclusion of these studies resulted in the odds ratio meta–estimate of 2.74 (95% CI 1.59–4.71). Two studies [20] (Rath, unpublished) were considered to be “low quality”. After excluding them, the final odds ratio meta–estimate was 2.79 (95% CI 2.19–3.55).

### Low birth weight

The six included studies used birth weight <2.5 kg to define low birth weight. One study [21] from Denmark used a definition of <3.0 kg, thus it was not included in the meta–analysis. Two hospital–based studies [16,25] [16,25] from industrialised countries reported significant associations between low birth weight and RSV–associated ALRI using multivariable analysis. Four additional studies [24,26] (Rath, unpublished; Zar, unpublished), one of which (Zar, unpublished) was from a developing country, reported odds ratios using univariable analysis. When data from these studies were combined with the data from studies using multivariable analysis, the overall odds ratio meta–estimate was 1.37 (95% CI 0.85–2.21). After excluding one study with “low quality” (Rath, unpublished), the final meta–estimate was 1.91 (95% CI 1.45–2.53).

### Being male

Five hospital–based studies [14,18,23,25,28] and one community–based study (Rasmussen, unpublished), reported associations between being male and RSV–associated ALRI using multivariable analysis. Only two of them reported non–significant associations [18] (Rasmussen, unpublished). The overall odds ratio meta–estimate was 1.32 (95% CI 1.24–1.40). Seven additional studies [16,19,24,26,30] (Rath, unpublished; Zar, unpublished), two of which were from developing countries, reported the odds ratios using univariable analysis. Two studies [19] (Rath, unpublished) were based on hospital inpatients and outpatients and another one (Zar, unpublished) was based on active community ascertainment. The inclusion of these studies did not alter the odds ratio meta–estimate substantially (OR 1.21, 95% CI 1.12–1.32). Excluding one “low–quality” study (Rath, unpublished), the final meta–estimate was 1.23 (95% CI 1.13–1.33).

### Siblings

Six hospital–based studies [14,21,23,24,28,29], one of which was from a developing country [23], reported associations between siblings (mention of siblings or other children living in the house) and RSV–associated ALRI using multivariable analysis. Only one of them reported a non–significant association [21]. The overall odds ratio meta–estimate was 1.53 (95% CI 1.20–1.95). Six additional studies [6,22,26,30] (Rasmussen, unpublished; Zar, unpublished), one of which was from an industrialised country [26], reported odds ratios for siblings and RSV–associated ALRI using univariable analysis. Three studies [22] (Rasmussen, unpublished; Zar, unpublished) were based on active community ascertainment and reported risk estimates for RSV–associated ALRI. The inclusion of these studies did not have any substantial effect on the odds ratio meta–estimate (OR 1.62, 95% CI 1.34–1.95). One study [22] was denoted as “low quality”. The final meta–estimate was 1.60 (95% CI 1.32–1.95) after excluding this study.

### Maternal smoking

Four hospital–based studies [14,18,21,28], all of which were from industrialised countries, reported associations between maternal smoking during pregnancy and hospitalised RSV–associated ALRI using multivariable analysis. Only one of them reported a non–significant association [18]. The overall odds ratio meta–estimate was 1.34 (95% CI 1.26–1.42). Three additional studies [19,29] (Zar, unpublished) reported data using univariable analysis. Two community–based studies from the Netherlands and South Africa [19] (Zar, unpublished) reported non–significant odds ratios for maternal smoking and RSV–associated ALRI and another hospital–based study from Denmark [29] reported a significant odds ratio. The inclusion of these studies resulted in a similar odds ratio meta–estimate of 1.36 (95% CI 1.24–1.50). All studies were considered to be of “good quality”.

### History of atopy

One hospital–based study [28] from Denmark reported a significant association between history of atopy (positive family history of asthma or atopy) and hospitalised RSV–associated ALRI using multivariable analysis. Four additional studies [19,26,30] (Zar, unpublished), two of which were from developing countries [30] (Zar, unpublished), reported the odds ratios using univariable analysis. One study [19] was based on both inpatient and outpatient settings in hospital and another one (Zar, unpublished) was community–based study. The overall odds ratio meta–estimate was 1.47 (95% CI 1.16–1.87). No studies had “low quality”.

### Low parental education

Definitions of low parental education varied among the included studies. Four studies [14,19,23] (Rasmussen, unpublished), two of which were from developing countries [23] (Rasmussen, unpublished), reported associations between low parental education (no parent having bachelor’s degree, <11 or <12 years maternal education, primary or no schooling) and RSV–associated ALRI using multivariable analysis; two of them reported significant associations. One study [19] was based on both inpatient and outpatient settings in hospital. One study (Rasmussen, unpublished) was a community–based study. The overall odds ratio meta–estimate was 1.23 (95% CI 0.73–2.09). Three additional studies [6,22] from developing countries reported odds ratios for low parental education (≤12 grade or primary or no schooling) and RSV–associated ALRI using univariable analysis. Two studies [22] were based on active community ascertainment and reported RSV–associated ALRI. The inclusion of these three studies resulted in a slightly higher odds ratio meta–estimate of 1.77 (95% CI 0.91–3.46). After excluding one low–quality study [22], the meta–estimate was 1.40 (95% CI 0.94–2.08) in the end.

### Passive smoking

Three hospital–based studies and one community–based study reported the associations between passive smoking (smokers in the house) and RSV–associated ALRI using multivariable analysis with the meta–estimate 1.40 (95% CI 0.65–3.00) [29,30] (Rasmussen, unpublished; Singleton, unpublished). Only one study from Denmark [29] reported a significant association. Five additional studies [6,22,24,26] (Zar, unpublished), two of which were from industrialised countries [24,26], reported odds ratios for passive smoking and RSV–associated ALRI using univariable analysis. Only two studies reported significant associations [6,24]. Two studies [22] (Zar, unpublished) were based on active community ascertainment. After combining studies using multivariable analysis and univariable analysis, the odds ratio meta–estimate was 1.23 (95% CI 0.95–1.60). One study [22] was “low quality”. After excluding this study, the final meta–estimate was 1.29 (95% CI 0.96–1.73).

### Daycare center attendance

One hospital–based study [28] from Denmark reported a significant association between daycare center attendance and hospitalised RSV–associated ALRI using multivariable analysis (OR 1.40, 95% CI 1.15–1.70). One study [19] from the Netherlands based on both inpatient and outpatient settings in hospital reported a non–significant association between daycare center attendance and RSV–associated ALRI using multivariable analysis (OR 5.80, 95% CI 0.76–44.4). One community–based study (Zar, unpublished) from South Africa also reported a non–significant association using univariable analysis. Overall, the odds ratio meta–estimate was 1.61 (95% CI 0.98–2.64). All studies were of “good quality” and were included in the final analysis.

### Indoor air pollution

Three hospital–based studies [30,34] (Singleton, unpublished) from Alaska and Gambia reported associations between indoor air pollution (woodstove in household) and hospitalised RSV–associated ALRI using multivariable or univariable analysis. Another study [27] from Guatemala based on both inpatient and outpatient settings in hospital reported a non–significant association using multivariable analysis (OR 0.76, 95% CI 0.42–1.16). A further two studies (Rasmussen, unpublished; Zar, unpublished) based on active community ascertainment from Pakistan and South Africa also reported non–significant associations using univariable analysis. Overall, the meta–estimate of odds ratio was 0.86 (95% CI 0.57–1.31). One study [34] was considered as having “low quality”, thus after excluding this study, the final meta–estimate was 0.81 (95% CI 0.42–1.57).

### No breastfeeding

Three hospital–based studies [6] (Singleton, unpublished; Zar, unpublished) from developing countries reported associations between no breastfeeding and RSV–associated ALRI. Only one of them [6] reported a significant association based on univariable analysis. These three studies all had “good quality” and the overall meta–estimate of odds ratio was 2.24 (95% CI 1.56–3.20). Another four studies [19,26,29] (Zar, unpublished), three from industrialised countries, reported odds ratios for lack of breastfeeding (no breastfeeding for first 14 days, <3 months breastfeeding or lack of exclusive breastfeeding) and RSV–associated ALRI. Only one study [29] reported a significant odds ratio based on multivariable analysis. One study [19] was based on both inpatient and outpatient settings in hospital and another one (Zar, unpublished) was community–based. Since these four studies used substantially different definitions for breastfeeding, meta–analysis was not carried out.

### Crowding

Included studies used varied definitions for crowding. Four studies [6] (Rath, unpublished; Singleton, unpublished; Zar, unpublished) reported associations between crowding (>7 persons in household) and RSV–associated ALRI. One study (Rath, unpublished) from Germany only had 5 children with crowding (5 in case group and 0 in control group). The prevalence of crowding is too small to generate a reliable estimate, thus this study was not included in analysis. One of them (Singleton, unpublished) reported the association using multivariate analysis. One study (Zar, unpublished) was community–based and the other two were hospital–based. These three studies all had “good quality”. Overall, the meta–estimate of the odds ratios was 1.94 (95% CI 1.29–2.93). Other studies used substantially different case definitions and, for these, meta–analysis was not done. Two studies [6,34] from Alaska presented significant associations between crowding (defined as ≥2 persons/room in household and an increase of 20% of households >1.5 persons/room) and hospitalised RSV–associated ALRI. One hospital–based study [30] from Gambia also reported a significant association using the definition of ≥10 people living in the household. Two community–based studies from Kenya [22] and Pakistan (Rasmussen, unpublished) reported non–significant associations with definitions of ≥3 siblings/room or >7 persons/room.

### Multiple births

Only one study [18] from New Zealand reported a non–significant association between multiple births (twins or triplets) and hospitalised RSV–associated ALRI using multivariable analysis. Two additional studies reported non–significant odds ratios using univariable analysis. One study from Spain [16] presented the association for multiple births and hospitalised RSV–associated ALRI while another study from Kenya [22] was based on active community–based case ascertainment. After combining these three studies, the odds ratio meta–estimate was 1.41 (95% CI 0.98–2.03). However, one study [22] was considered as “low–quality” and thus no meta–estimate was available after excluding this study.

### HIV

Three hospital–based studies [20,32,33] from South Africa reported significant associations between HIV (confirmed presence of HIV infection in child) and RSV–associated ALRI. One of them reported an age–adjusted association and provided data for two years separately [33]. The overall meta–estimate of odds ratio was 3.74 (95% CI 2.47–5.66). Two of them [20,32] were considered to be of “low quality”. Thus no meta–estimate was available after we excluded these two “low–quality” studies.

### Malnutrition

Only three studies were included. Two community–based studies from Kenya [22] and South Africa (Zar, unpublished) reported non–significant associations between malnutrition (weight for age ≤2 standard deviations) and RSV–associated ALRI using univariable analysis (OR 1.28, 95% CI 0.75–2.21) and 1 (95% CI 0.4–2.9). Another hospital–based study [23] from the Philippines reported a significant association between measures less than or equal to median growth (weight for age) and hospitalised RSV–associated ALRI using multivariable analysis (OR 1.34, 95% CI 1.02–1.76).

### Altitude

Only one hospital–based study [15] from Colorado reported a significant association between high altitude and hospitalised RSV–associated ALRI using multivariable analysis, stratified by age and control group. The odds ratio of RSV–associated hospitalised ALRI among infants at high altitude (>2500 m) compared to moderate altitude (1500–2500 m) was 1.30 while it was 1.22 when compared to low altitude (<1500 m). Also, the odds ratio among children aged 1–4 years old in high altitude was 1.80 when compared to moderate altitude and 1.62 when compared to low altitude.

### Previous illness

One hospital–based study [26] from Italy reported a significant association between no previous RSV infections and hospitalised RSV–associated ALRI using univariable analysis (OR 1.85, 95% CI 1.02–3.36). Another community–based study from South Africa (Zar, unpublished) reported a significant association between previous history of ALRI and RSV–associated ALRI using univariable analysis (OR 3.9, 95% CI 1.2–12.5).

### Lack of plumbed water (available within the household)

Two hospital–based studies [34,35] from Alaska reported significant associations between lack of plumbed water or low proportion in–home water service (<80%) and hospitalised RSV–associated ALRI (OR 1.45, 95% CI 1.19–1.78 and OR 2.81, 95% CI 2.42–3.26 respectively). However, both studies were considered to be of “low–quality”. Another study from Gambia [30] reported “tap in compound” compared to other water sources and the adjusted OR was 1.75 (95% CI 0.85–3.60). This number was converted to be comparable to those two studies mentioned above.

### DISCUSSION

Our study presents the most up–to–date and comprehensive report of the strength of association between various socio–demographic risk factors and RSV–associated ALRI in children younger than five years old. After excluding “low–quality” studies, we identified a total of 18 putative risk factors, of which 8 (prematurity, low birth weight, being male, siblings, maternal smoking, history of atopy, no breastfeeding and crowding ≥7 persons in household) were observed to be significantly associated with RSV–associated ALRI. Ten additional risk factors (low parental education, passive smoking, daycare center attendance, indoor air pollution, HIV, multiple births, malnutrition, higher altitude, previous illness and lack of plumbed water in the household) were also observed to have an association with RSV–associated ALRI in 1–3 studies. However, for some of these risk factors (eg, lack of breastfeeding, crowding), meta–analysis could not be performed to generate odds ratio meta–estimate as case definitions were substantially different or sufficient studies were not available (eg, HIV, multiple births). Therefore, the associations between these risk factors and RSV–ALRI require further study.

There was considerable variation among the 27 included studies (including “low–quality” studies). Nine [14,17,19,20,22,23,25,29] (and Rath, unpublished) were cohort studies; 11 [6,16,18,21,24,26,28,30] (and Rasmussen, unpublished; Singleton, unpublished; Zar, unpublished) were case-control studies; 6 [15,31-35] were cross-sectional studies; and 1 [27] was a randomized controlled trial. Most studies used questionnaires or interviews (of caretakers or parents) to gather information on various risk factors, which could be a source of several biases, such as response bias, recall bias, interviewer bias and misclassification bias. Other potential biases also existed. For example, there could be follow–up bias in cohort studies. Among eleven case-control studies, only 7 [6,21,24,28,30] (Rasmussen, unpublished; Zar, unpublished) selected a control group matched by date of birth and/or sex and/or location of residence, which could introduce substantial bias in the selection of controls in studies which did not use matched control groups.

There were substantial differences with regards to the number of confounders adjusted in each study. Seven studies [15,18,23,25,27,28] (and Singleton, unpublished) used multivariable analysis to adjust for all other risk factors of interest investigated in the same study. Some also adjusted for age at third dose of pneumococcal conjugate vaccine, age at risk and weight for age *z*–score at first vaccination [23], or population distribution of education level, households that were living below poverty level and race [15]. One study reported age adjusted relative risk [33]. Four studies [20,29,32,33] also reported concurrent bacteraemia or coinfection with other viruses. Another 7 studies used univariable analysis, and 12 studies reported estimates using both multivariable and univariable analysis.

The quality score of each study obtained from modified GRADE scoring system varied from 2.5 to 11 with a mean of 7.6. There were 7 studies with “low quality” (quality score ≤6.25). Most of them were not designed as case–control studies, did not consider biases within the research, did not take into account of potential confounders or reported estimates using multivariable analysis. A sensitivity analysis was carried out to include these “low–quality” studies. The meta–estimate OR from sensitivity analysis did not differ substantially from the analysis where only studies with quality scores >6.25 were included (Appendix S8 in the **Online Supplementary Document(Online Supplementary Document)**). However, this quality assessment tool did not address all aspects related to study quality since we only looked into seven of these: study design, quality of control group, sample size, analysis method, bias, confounding factors and geographical spread of studies. More detailed and appropriate quality assessment tools should be applied and studies with higher quality would be needed to generate more reliable results.

It is noteworthy that there was substantial heterogeneity in the specific definition for a risk factor in each of the included studies, which limited our analysis. For example, six studies used a definition of birthweight <2.5 kg to define low birth weight, while one study [21] used a higher threshold–birthweight <3.0 kg, and was therefore excluded from the meta–analysis. Nine studies defined prematurity as gestational age <37 weeks, while three studies [14,20,26] used gestational age <36 weeks and another one [29] used <38 weeks. After excluding “low–quality” studies and these four studies using different definitions of prematurity, the meta–estimate of the association between prematurity (gestational age <37 weeks) and RSV–associated ALRI was 1.96 (95% CI 1.44–2.67), which was similar to the alternative estimate 1.98 (95% CI 1.56–2.52) when all studies irrespective of quality scores were included. Only one study (Zar, unpublished) reported that prematurity was determined using ultrasonography. Seven studies defined low parental education using five different definitions–no parent having bachelor’s degree [19], 1–7 years of education or no schooling for primary caretaker [22] (Zar, unpublished), 1–5 years of education or no schooling for parents (Rasmussen, unpublished), <12 years maternal education [6,14] and <10 years maternal education [23]. Since there were insufficient studies in each category, we did not conduct a subgroup meta–analysis. Similarly crowding was defined using substantially different definitions in the included studies: >7 persons living in household [6] (Singleton, unpublished; Zar, unpublished), ≥10 persons in household [30], ≥2 persons per room [6] (Zar, unpublished), ≥3 siblings less than 6 years old sleeping in the same room [22], >7 persons sleeping per room (Rasmussen, unpublished), an increase of 20% in number of households >1.5 persons/room [34]. Therefore, once again, we did not conduct a subgroup meta–analysis in this instance except for the definition of >7 persons living in household. The substantial variability in reporting definitions for the same risk factor require that standardised definitions should be proposed for future studies, which will improve the comparability of these studies.

Furthermore, there was variation in the age groups of participants included in each study. Only six studies included children younger than five years old [20,23,30,33] (Rath, unpublished), and 21 studies included children in narrower age bands (eg, 0–11months, 0–18 months, 0–23 months). Thirteen studies focused on children younger than two years old, among which, six studies included only infants (0–11 months) [14,17,19,29,34,35]. Since data from different age groups were pooled together, and RSV is predominantly an infection in children aged below 2 years [5], we may have overestimated the association between various risk factors and RSV–associated ALRI in children aged 0–59 months.

Also, the sample size of each study varied considerably. We only included studies with sample size greater than 50, as specified in our eligibility criteria. However, among the 27 included studies, the sample size varied from 68 (Singleton, unpublished) to 835 060 [33]. This is reflected in the wide confidence intervals of the ORs for some studies with small sample size, indicating less precise estimates.

Another limitation is that we did not have access to individual patient data on risk factors for RSV–associated ALRI. Further research should focus on obtaining individual patient data from previous studies or ongoing studies, such as multi–center Pneumonia Etiology Research for Child Health (PERCH) project. With these patient level data, we could have a better understanding about the role of each risk factor in RSV–associated ALRI (particularly with regard to prematurity) and adjust for possible confounders in a pooled analysis.

The definitions of some risk factors were similar or the same as those reported in a review [36] investigating risk factors for severe ALRI (for which etiology was not further specified), which indicates that pneumonia and RSV–associated ALRI do share a few socio–demographic risk factors which are amenable to interventions, such as maternal smoking, passive smoking and no breastfeeding. Appendix S9 in the **Online Supplementary Document(Online Supplementary Document)** shows the comparison of strength of association of risk factors identified in both reviews. The strength of association between risk factors and severe ALRI was generally slightly stronger than the corresponding ones in RSV–associated ALRI. Several risk factors were only investigated for severe ALRI, such as incomplete immunization, vitamin D deficiency, anemia, zinc deficiency, birth interval, birth order, and vitamin A deficiency, while some risk factors were only explored for RSV–associated ALRI (siblings, history of atopy, multiple births, high altitude, lack of plumbed water in the household).

Compared to the previous review [8] conducted over one decade ago, this review presented an overview of a larger number and more recent studies investigating more risk factors associated with RSV and summarized the findings using meta–analysis. Both reviews shared similar results for some risk factors, such as being male, crowding/siblings and day care attendance. Also, we provided more evidence for some risk factors which had an unclear role with regard to RSV (passive smoking, low parental education). Additionally, we identified more risk factors associated with RSV which were not available in previous review due to insufficient evidence (prematurity, low birth weight, maternal smoking, history of atopy, indoor air pollution, no breastfeeding). However, race/ethnicity, age of acquisition of RSV as well as birth during the first half of RSV season, which were investigated in previous review, were not evaluated in this review because no recent relevant studies were found. Moreover, several risk factors which were reported in some studies were not included in this search strategy or in the analysis, such as, siblings’ death, parents’ nationality, parents’ occupation, their roles also remained unknown [30].

Further research on this topic should identify, seek access to and analyze additional unpublished RSV data sets to further improve the precision of these estimates. This should include, where possible, investigation of possible association with risk factors which have been reported to show association with (all cause) ALRI: incomplete immunization, vitamin D deficiency, anemia, zinc deficiency, birth interval, birth order, and vitamin A deficiency.

## CONCLUSION

RSV is a major cause of hospital admission and mortality among young children, especially in developing countries [5]. Our study assessed the role of putative socio–demographic risk factors for RSV–associated ALRI. Many of these risk factors are similar to those that have been identified for (all cause) ALRI and thus, in addition to the potential future impact of novel RSV vaccines currently under development and evaluation, national action against ALRI risk factors as part of national control programmes [37] can be expected to reduce burden of disease from RSV. The evidence generated from this study could be used to model the global, regional and national estimates of RSV–associated ALRI. Since some risk factors are preventable, policy makers and public health practitioners could develop targeted interventions to decrease the prevalence of these risk factors in order to reduce RSV–associated ALRI disease burden. However, this evidence base is limited by paucity of data. Therefore, large scale, high quality multivariable studies should be conducted on a priority basis to better understand the role of each individual risk factor for RSV–associated ALRI in diverse settings.
