# Supplementary material for: Risk factors for respiratory syncytial virus associated with acute lower respiratory infection in children under five years: Systematic review and meta–analysis
Source: J Glob Health. 2015 Dec 8;5(2):020416. doi: 10.7189/jogh.05.020416 (PMC4676580; doi:10.7189/jogh.05.020416)
Supplement: Online Supplementary Document [file jogh-05-020416-s001.pdf]

Online Supplementary Document

Shi et al. Risk factors for respiratory syncytial virus associated with acute lower respiratory infection in children under five years: Systematic review and meta–analysis

J Glob Health 2015;5:020416

Appendix 1: Search strategy

Medline

1. exp Respiratory Syncytial Viruses/ or exp Respiratory Syncytial Virus Infections/ or RSV.mp. or exp Respiratory Syncytial Virus, Human/

2. respiratory syncytial virus*.mp.

3. 1 or 2

4. pneumonia.mp. or exp Pneumonia/ or exp Pneumonia, Viral/

5. bronchiolitis.mp. or exp Bronchiolitis/ or exp Bronchiolitis, Viral/

6. exp Respiratory Tract Infections/ or respiratory infection*.mp.

7. exp Respiratory Tract Diseases/ or respiratory disease*.mp.

8. 4 or 5 or 6 or 7

9. risk factor*.mp. or exp Risk Factors/

10. low birth weight.mp. or exp Infant, Low Birth Weight/

11. breast feeding.mp. or exp Breast Feeding/

12. exp Crowding/ or crowd*.mp.

13. indoor air pollution.mp. or exp Air Pollution, Indoor/

14. exp Malnutrition/ or malnutrition.mp.

15. exp Measles Vaccine/ or measles vaccin*.mp.

16. exp HIV/ or HIV.mp.

17. exp Infant, Premature/ or prematur*.mp.

18. asthma.mp. or exp Asthma/

19. exp Education/ or education.mp.

20. exp Altitude/ or altitude.mp.

21. child care.mp. or exp Child Care/

22. day care.mp. or exp Day Care/

23. atop*.mp.

24. exp Tobacco Smoke Pollution/ or tobacco smok*.mp.

25. hygiene.mp. or Hygiene/

26. water.mp. or Water/ or Water Supply/

27. 9 or 10 or 11 or 12 or 13 or 14 or 15 or 16 or 17 or 18 or 19 or 20 or 21 or 22 or 23 or 24 or 25 or 26

28. 3 and 8

29. 27 and 28

30. limit 29 to (humans and yr="1995 -Current" and ("all infant (birth to 23 months)" or "newborn infant (birth to 1 month)" or "infant (1 to 23 months)" or "preschool child (2 to 5 years)"))

Embase

1. exp respiratory syncytial virus infection/ or RSV.mp. or exp Respiratory syncytial pneumovirus/

2. respiratory syncytial virus*.mp.

3. exp community acquired pneumonia/ or exp pneumonia/ or exp virus pneumonia/ or exp infectious pneumonia/ or pneumonia.mp.

4. bronchiolitis.mp. or exp bronchiolitis/ or exp viral bronchiolitis/

5. exp lower respiratory tract infection/ or exp respiratory tract infection/ or respiratory infection*.mp.

6. exp respiratory tract disease/ or respiratory disease*.mp.

7. 1 or 2

8. 3 or 4 or 5 or 6

9. exp risk factor/ or risk factor*.mp.

10. low birth weight.mp. or exp low birth weight/

11. breast feeding.mp. or exp breast feeding/

12. exp crowding/ or crowd*.mp.

13. indoor air pollution.mp. or exp indoor air pollution/

14. exp malnutrition/ or malnutrition.mp.

15. exp measles vaccine/ or measles vaccin*.mp.

16. HIV.mp. or exp Human immunodeficiency virus/

17. exp prematurity/ or prematur*.mp.

18. exp asthma/ or asthma.mp.

19. education.mp. or exp education/

20. altitude.mp. or exp altitude/

21. child care.mp. or exp child care/

22. day care.mp. or exp day care/

23. atop*.mp.

24. exp smoking/ or exp tobacco smoke/ or exp passive smoking/ or tobacco smok*.mp.

25. water.mp. or water/ or water supply/

26. hygiene/ or hygiene.mp.

27. 9 or 10 or 11 or 12 or 13 or 14 or 15 or 16 or 17 or 18 or 19 or 20 or 21 or 22 or 23 or 24 or 25 or 26

28. 7 and 8 and 27

29. limit 28 to (human and yr="1995 -Current" and (infant or preschool child <1 to 6 years>))

Global Health

1. exp human respiratory syncytial virus/ or rsv.mp.
2. respiratory syncytial virus*.mp.
3. 1 or 2
4. exp community acquired pneumonia/ or pneumonia*.mp. or exp pneumonia/
5. bronchiolitis.mp. or exp bronchiolitis/
6. respiratory infection*.mp.
7. exp respiratory diseases/ or respiratory disease*.mp.
8. 4 or 5 or 6 or 7
9. exp risk factors/ or risk factor*.mp.
10. low birth weight.mp. or exp low birth weight infants/
11. exp premature infants/ or exp prematurity/ or prematur*.mp.
12. exp asthma/ or asthma.mp.
13. exp atopy/ or atop*.mp.
14. exp education/ or education.mp.
15. exp breast feeding/ or breast feeding.mp.
16. exp altitude/ or altitude.mp.
17. exp malnutrition/ or malnutrition.mp.
18. exp child care/ or child care.mp.
19. exp day care/ or day care.mp.
20. exp Crowding/ or crowd*.mp.
21. exp indoor air pollution/ or indoor air pollution.mp.
22. exp passive smoking/ or exp tobacco smoking/ or tobacco smok*.mp.
23. HIV.mp. or exp human immunodeficiency viruses/
24. measles vaccin*.mp.
25. exp water supply/ or water.mp. or exp water/
26. exp hygiene/ or hygiene.mp.
27. 9 or 10 or 11 or 12 or 13 or 14 or 15 or 16 or 17 or 18 or 19 or 20 or 21 or 22 or 23 or 24 or 25 or 26
28. 3 and 8 and 27
29. limit 28 to yr="1995 -Current"

LILACS (AMRO/PAHO)

Respiratory syncytial virus and risk factor* in All Indexes

Appendix 2: List of all various definitions of risk factors for RSV associated ALRI in the included studies

Risk factor	Definition
**Prematurity**	Gestational age <37 weeks (±1 week)<br>Gestational age <33 weeks
**Low birth weight**	Birth weight <2.5 kg (or <3.0 kg)
**No/lack of exclusive breastfeeding**	No breastfeeding<br>No or <3 months breastfeeding<br>No breastfeeding for first 14 days<br>Lack of exclusive breastfeeding
**Multiple births**	Twins or triplets
**Gender**	Male
**History of atopy**	Positive family history of asthma or atopy
**Low parental education**	No parent having bachelor's degree<br>Education of primary caretaker: 1-7 years or no schooling<br><12 years maternal education<br><11 years maternal education
**Siblings**	Mention of siblings or other children living in the household
**Passive smoking**	Smokers in the household
**Maternal smoking**	Maternal smoking during pregnancy
**Daycare centre attendance**	Attendance at daycare centre
**Crowding**	>7 persons in household<br>≥10 persons in household<br>≥2 persons per room<br>≥3 siblings less than 6 years old sleeping in same room<br>>7 persons sleeping per room<br>An increase of 20% of households >1.5 persons/room
**Malnutrition**	Weight for age ≤2 standard deviations (SD)<br>Growth ≤median measures (weight for age)
**Indoor air pollution**	Use of biomass fuels for cooking or a description of indoor smoke
**HIV**	Confirmed presence of HIV infection in child
**Previous illness**	Previous history of ALRI/RSV
**Altitude**	High altitude
**Lack of plumbed water**	Lack of plumbed water in the household, low in-home water service or using other water sources rather than tap in compound

Appendix 3: Modified GRADE scoring system

Criteria	Categorisation	Score
Design	Cohort study	+2 point
	Case control study	+1 points
	Cross sectional study	0.5 points
	Randomised control trial	0 points
Quality of control group	Good (matched to cases)	+2 points
	Average (not matched, but some descriptions about control)	+1 point
	Poor (no further details)	0 point
Sample size	>500 subjects	+2 points
	300-500 subjects	+1 point
	<300 subjects	0 point
Analysis	Only multivariable	+2 points
	Part of analyses were based on multivariable	+1 point
	Only univariable	0 point
Bias	Good attempt to avoid bias (selection bias etc.)	+2 points
	Some attempt to avoid bias (selection bias)	+1 point
	No attempt to avoid bias	0 point
Confounding factors	All accounted for (analysis of each risk factor has taken into account of the other risk factors investigated in the same study)	+2 points
	Some accounted for (analyses of some risk factors have taken into account of the other risk factors investigated in the same study)	+1 point
	Not accounted for	0 point
Geographical spread of studies	Good geographical distribution	+2 points
	Limited geographical distribution	+1 point
	Studies in one region only (one area)	0 point

Appendix 4: Score of each study based on modified GRADE scoring system

Study	Design	Quality of control group	Sample size	Analysis	Bias	Confounding factors	Geographical spread of studies	Total
(Von Linstow et al., 2008)	+2	+2	0	+1	+1	+1	0	+7
(Stensballe et al., 2006)	+1	+2	+2	+2	+1	+1	+2	+11.5
(Houben et al., 2011)	+2	+1	0	+1	+2	+1	+1	+8
(Choudhuri et al., 2006)	+0.5	+1	+2	+2	0	+2	+2	+9.5
(Smith et al., 2011)	0	+1	+2	+2	+1	+1	+1	+8
(Okiro et al., 2008)	+2	+1	+1	+1	0	+1	0	+6
(Madhi et al., 2006)	+2	+1	+2	0	0	0	0	+5
(Rietveld et al., 2006)	+2	+1	+2	+2	0	+1	+2	+10
(Gouyon et al., 2013)	+2	+1	+1	0	+1	+1	+2	+8
(Weigl et al., 2001)	+0.5	+1	+2	0	0	0	+1	+4.5
(Paynter et al., 2014)	+2	+1	+2	+2	+1	+2	+1	+11
(Reeve et al., 2006)	+1	+2	+2	+1	+2	+1	+1	+10
(Boyce et al., 2000)	+2	+1	+2	+1	0	+1	+2	+9
(Nielsen et al., 2003)	+1	+2	+2	+1	+2	+1	+1	+10
(Cilla et al., 2006)	+1	+1	+2	+1	+1	+1	0	+7
(Grimwood et al., 2008)	+1	+1	+2	+2	+1	+1	0	+8
(Bulkow et al., 2002)	+1	+2	+2	+1	+1	+1	+1	+9
(Weber et al., 1999)	+1	+2	+2	+1	+1	+1	+1	+9
(Rossi et al., 2007)	+1	+1	+1	+1	+1	+1	+1	+7
(Singleton, unpublished)	+1	+2	0	+2	0	+1	+1	+7
(Rasmussen, unpublished)	+1	+2	0	+1	+2	+1	+1	+8

Study	Design	Quality of control group	Sample size	Analysis	Bias	Confounding factors	Geographical spread of studies	Total
(Madhi et al., 2000)	+0.5	0	+2	0	0	0	0	+2.5
(Moyes et al., 2013)	+0.5	0	+2	+2	0	+1	+1	+6.5
(Hennessy et al., 2008)	+0.5	0	+2	0	0	0	+1	+3.5
(Bruden et al., 2015)	+0.5	0	+2	+1	0	+1	+1	+5.5
(Zar, unpublished)	+2	+2	0	0	+2	+1	+2	+9
(Rath, unpublished)	+2	+1	+2	0	0	0	+1	+6

Appendix 5: Location of the 27 studies

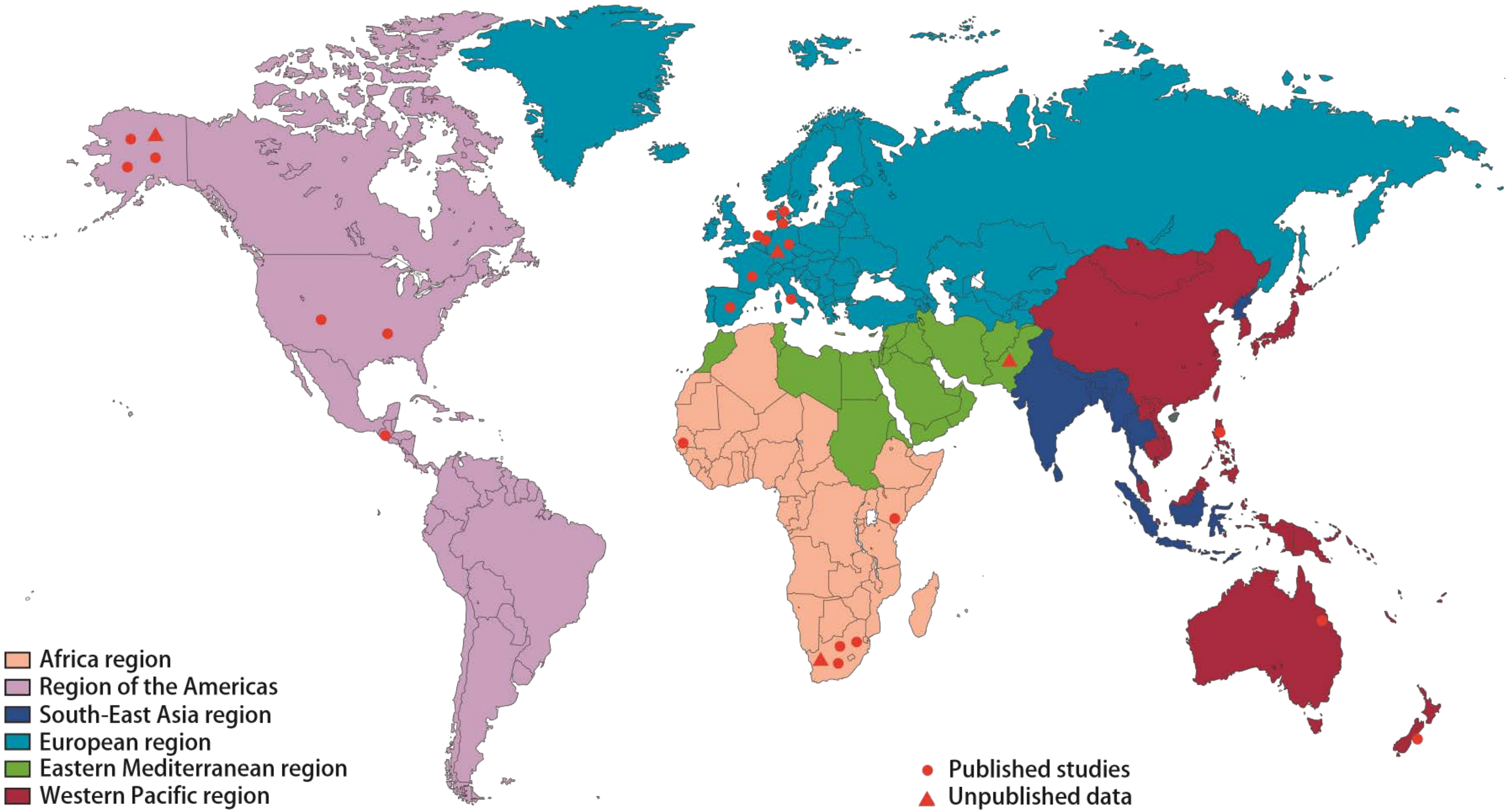

Appendix 6: Forest plots for meta-analysis of risk factors excluding "low-quality" studies

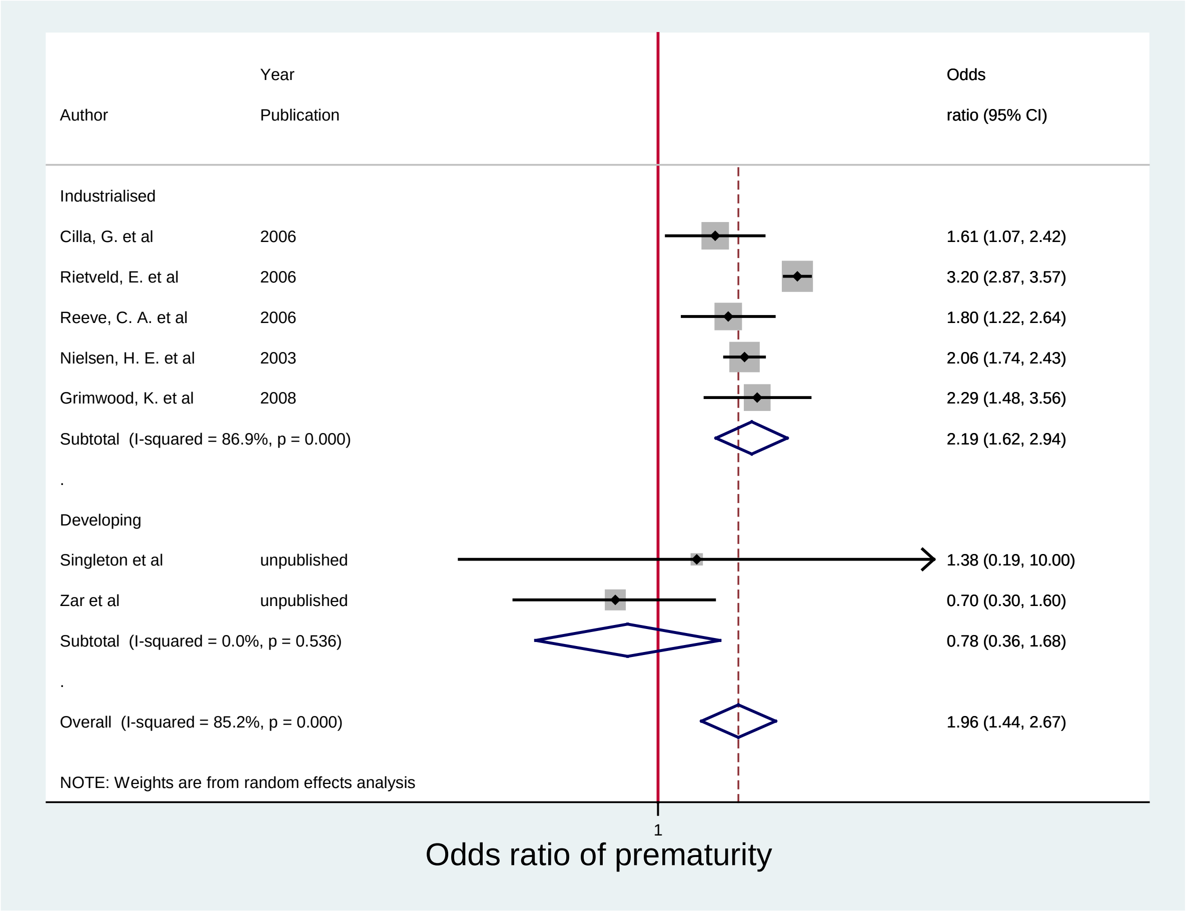

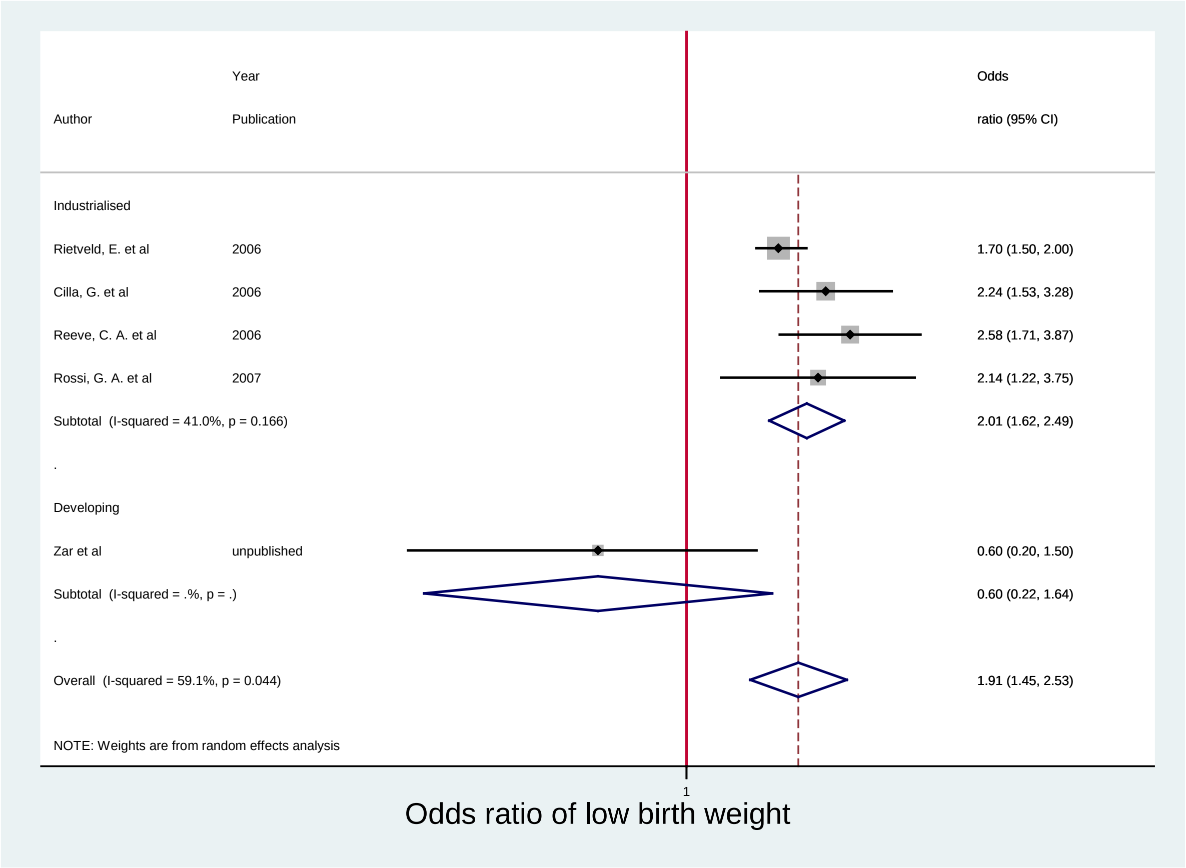
Author
Year
Publication
Odds
ratio (95% CI)
Industrialised
Rietveld, E. et al 2006 1.70 (1.50, 2.00)
Cilla, G. et al 2006 2.24 (1.53, 3.28)
Reeve, C. A. et al 2006 2.58 (1.71, 3.87)
Rossi, G. A. et al 2007 2.14 (1.22, 3.75)
Subtotal (I-squared = 41.0%, p = 0.166) 2.01 (1.62, 2.49)
Developing
Zar et al unpublished 0.60 (0.20, 1.50)
Subtotal (I-squared = .%, p = .) 0.60 (0.22, 1.64)
Overall (I-squared = 59.1%, p = 0.044) 1.91 (1.45, 2.53)
NOTE: Weights are from random effects analysis
1
Odds ratio of low birth weight

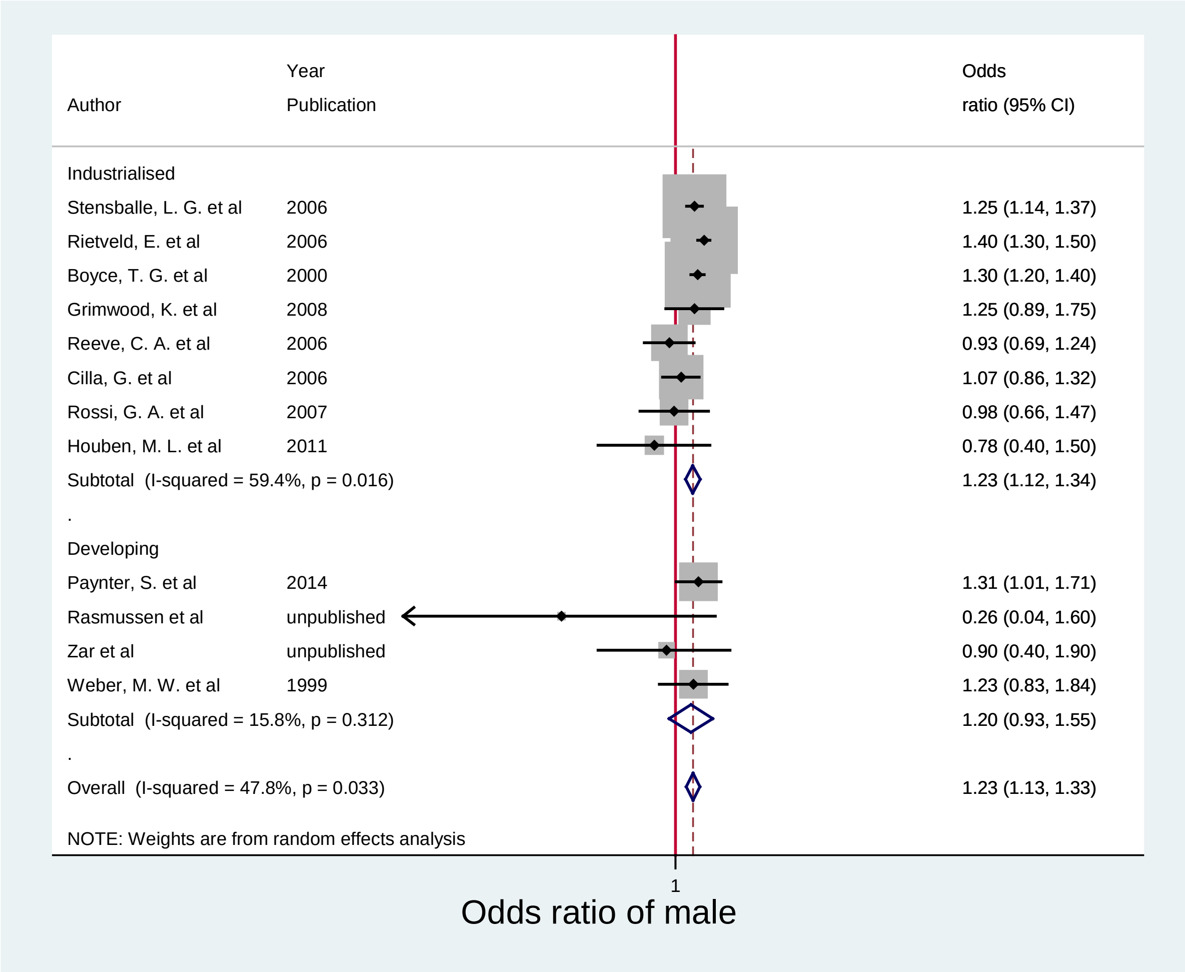
Author
Year Publication
Odds ratio (95% CI)
Industrialised
Stensballe, L. G. et al 2006 1.25 (1.14, 1.37)
Rietveld, E. et al 2006 1.40 (1.30, 1.50)
Boyce, T. G. et al 2000 1.30 (1.20, 1.40)
Grimwood, K. et al 2008 1.25 (0.89, 1.75)
Reeve, C. A. et al 2006 0.93 (0.69, 1.24)
Cilla, G. et al 2006 1.07 (0.86, 1.32)
Rossi, G. A. et al 2007 0.98 (0.66, 1.47)
Houben, M. L. et al 2011 0.78 (0.40, 1.50)
Subtotal (I-squared = 59.4%, p = 0.016) 1.23 (1.12, 1.34)
.
Developing
Paynter, S. et al 2014 1.31 (1.01, 1.71)
Rasmussen et al unpublished 0.26 (0.04, 1.60)
Zar et al unpublished 0.90 (0.40, 1.90)
Weber, M. W. et al 1999 1.23 (0.83, 1.84)
Subtotal (I-squared = 15.8%, p = 0.312) 1.20 (0.93, 1.55)
.
Overall (I-squared = 47.8%, p = 0.033) 1.23 (1.13, 1.33)
NOTE: Weights are from random effects analysis
1
Odds ratio of male

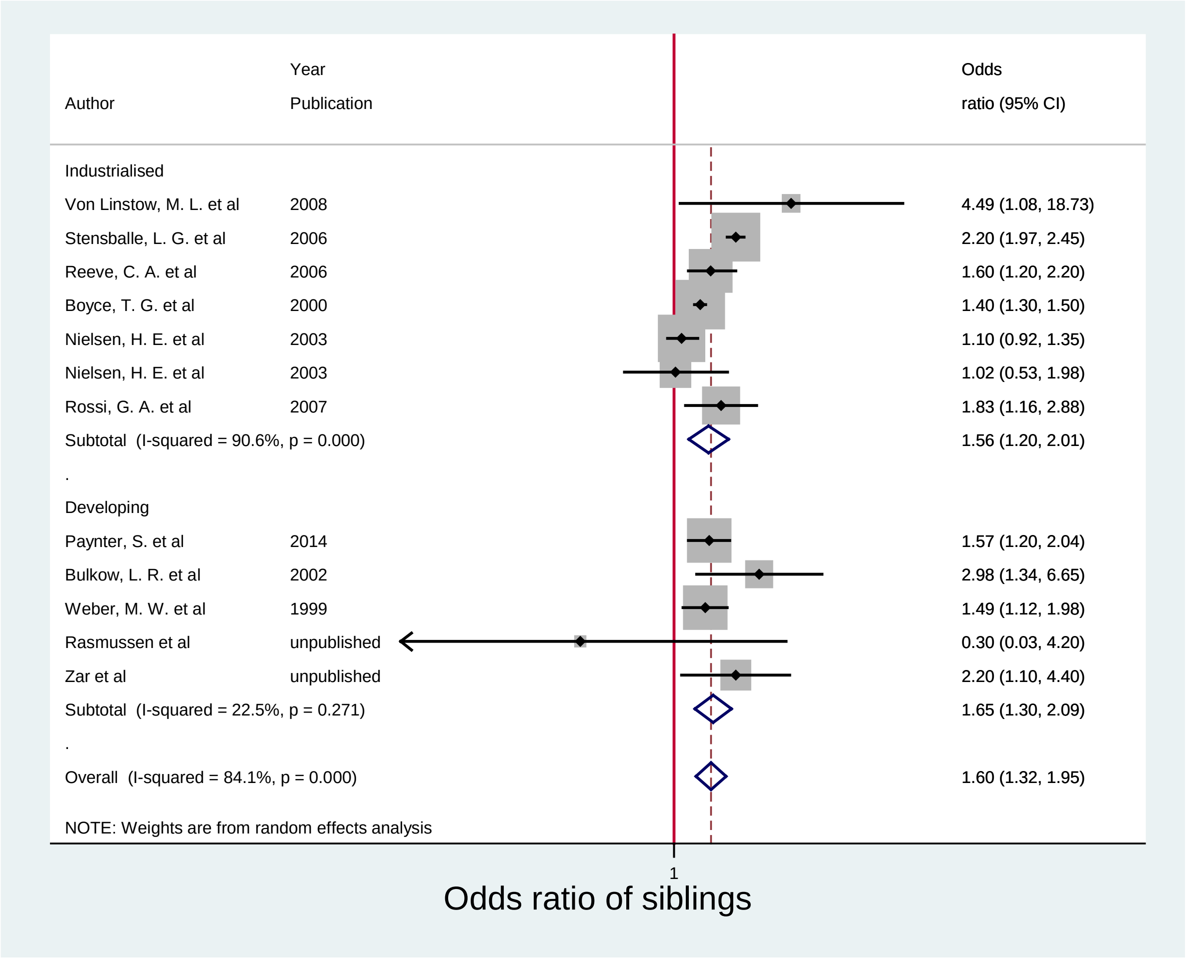
Author
Year
Publication
Odds
ratio (95% CI)
Industrialised
Von Linstow, M. L. et al 2008 4.49 (1.08, 18.73)
Stensballe, L. G. et al 2006 2.20 (1.97, 2.45)
Reeve, C. A. et al 2006 1.60 (1.20, 2.20)
Boyce, T. G. et al 2000 1.40 (1.30, 1.50)
Nielsen, H. E. et al 2003 1.10 (0.92, 1.35)
Nielsen, H. E. et al 2003 1.02 (0.53, 1.98)
Rossi, G. A. et al 2007 1.83 (1.16, 2.88)
Subtotal (I-squared = 90.6%, p = 0.000) 1.56 (1.20, 2.01)
.
Developing
Paynter, S. et al 2014 1.57 (1.20, 2.04)
Bulkow, L. R. et al 2002 2.98 (1.34, 6.65)
Weber, M. W. et al 1999 1.49 (1.12, 1.98)
Rasmussen et al unpublished 0.30 (0.03, 4.20)
Zar et al unpublished 2.20 (1.10, 4.40)
Subtotal (I-squared = 22.5%, p = 0.271) 1.65 (1.30, 2.09)
.
Overall (I-squared = 84.1%, p = 0.000) 1.60 (1.32, 1.95)
NOTE: Weights are from random effects analysis
1
Odds ratio of siblings

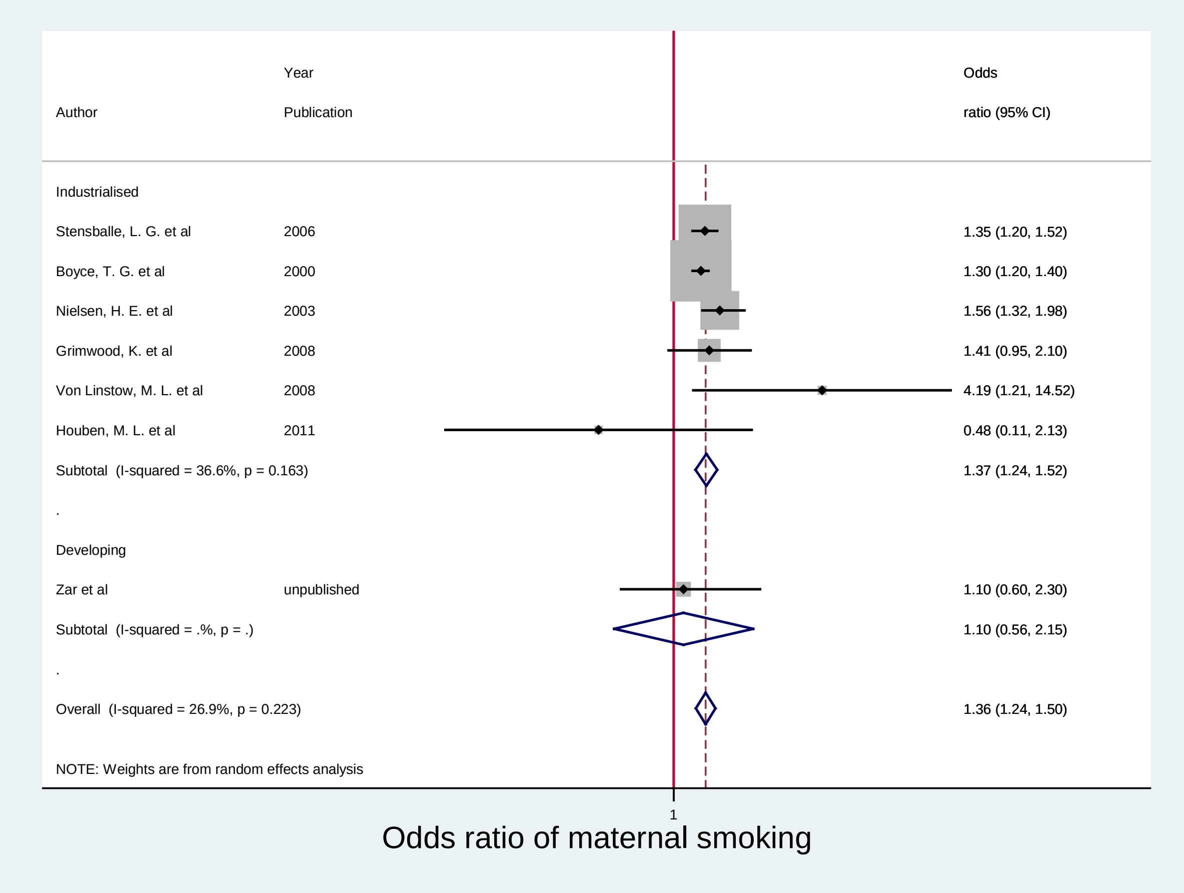
Author
Year
Publication
Odds
ratio (95% CI)
Industrialised
Stensballe, L. G. et al 2006 1.35 (1.20, 1.52)
Boyce, T. G. et al 2000 1.30 (1.20, 1.40)
Nielsen, H. E. et al 2003 1.56 (1.32, 1.98)
Grimwood, K. et al 2008 1.41 (0.95, 2.10)
Von Linstow, M. L. et al 2008 4.19 (1.21, 14.52)
Houben, M. L. et al 2011 0.48 (0.11, 2.13)
Subtotal (I-squared = 36.6%, p = 0.163) 1.37 (1.24, 1.52)
.
Developing
Zar et al unpublished 1.10 (0.60, 2.30)
Subtotal (I-squared = .%, p = .) 1.10 (0.56, 2.15)
.
Overall (I-squared = 26.9%, p = 0.223) 1.36 (1.24, 1.50)
NOTE: Weights are from random effects analysis
1
Odds ratio of maternal smoking

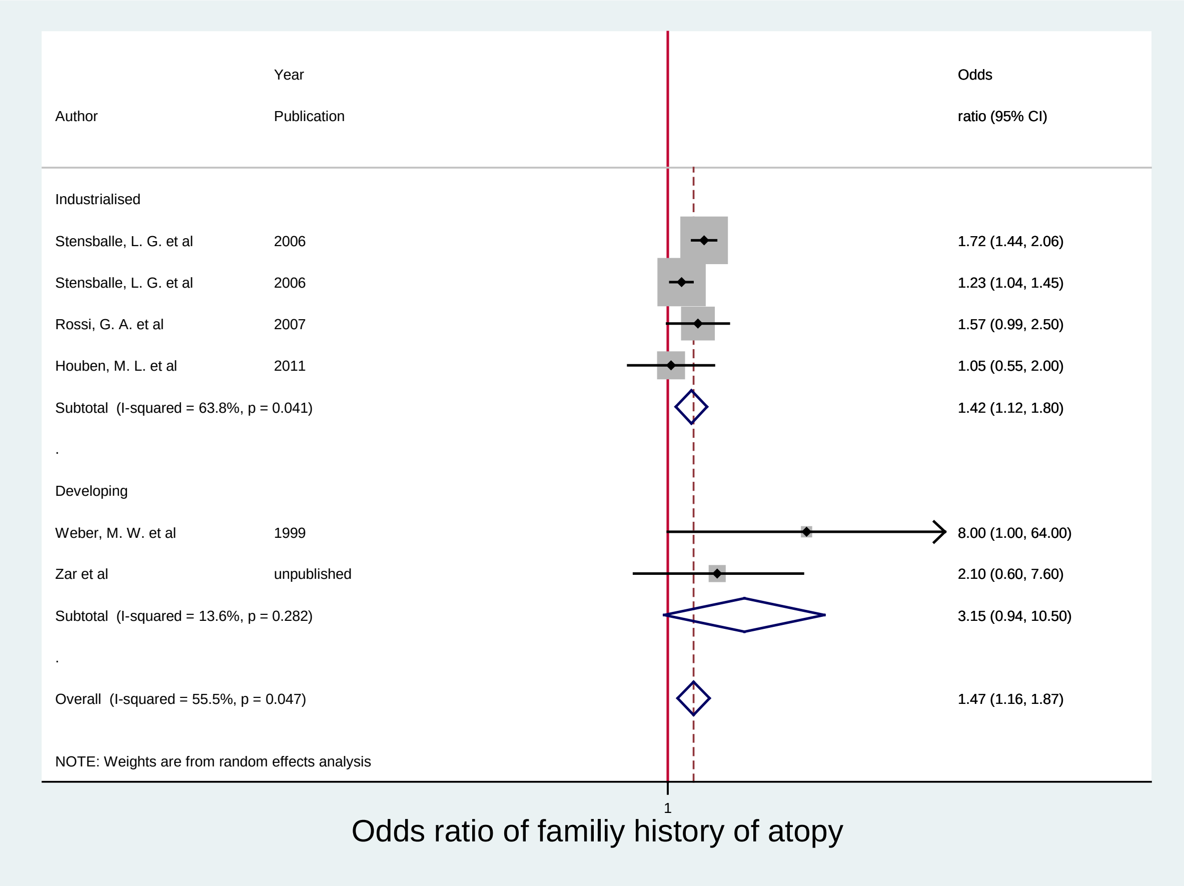
Author
Year
Publication
Odds
ratio (95% CI)
Industrialised
Stensballe, L. G. et al 2006 1.72 (1.44, 2.06)
Stensballe, L. G. et al 2006 1.23 (1.04, 1.45)
Rossi, G. A. et al 2007 1.57 (0.99, 2.50)
Houben, M. L. et al 2011 1.05 (0.55, 2.00)
Subtotal (I-squared = 63.8%, p = 0.041) 1.42 (1.12, 1.80)
.
Developing
Weber, M. W. et al 1999 8.00 (1.00, 64.00)
Zar et al unpublished 2.10 (0.60, 7.60)
Subtotal (I-squared = 13.6%, p = 0.282) 3.15 (0.94, 10.50)
.
Overall (I-squared = 55.5%, p = 0.047) 1.47 (1.16, 1.87)
NOTE: Weights are from random effects analysis
1
Odds ratio of familiy history of atopy

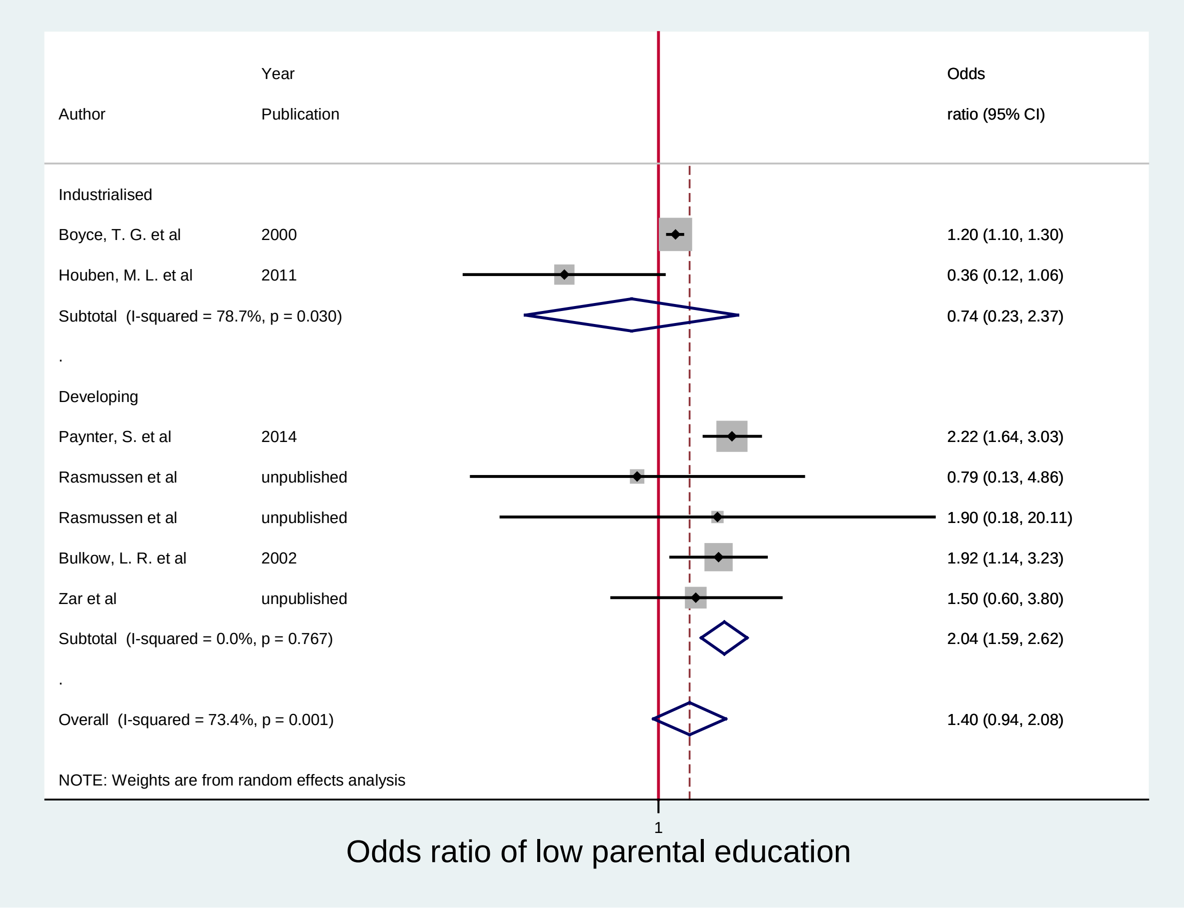
Author
Year
Publication
Odds
ratio (95% CI)
Industrialised
Boyce, T. G. et al 2000 1.20 (1.10, 1.30)
Houben, M. L. et al 2011 0.36 (0.12, 1.06)
Subtotal (I-squared = 78.7%, p = 0.030) 0.74 (0.23, 2.37)
.
Developing
Paynter, S. et al 2014 2.22 (1.64, 3.03)
Rasmussen et al unpublished 0.79 (0.13, 4.86)
Rasmussen et al unpublished 1.90 (0.18, 20.11)
Bulkow, L. R. et al 2002 1.92 (1.14, 3.23)
Zar et al unpublished 1.50 (0.60, 3.80)
Subtotal (I-squared = 0.0%, p = 0.767) 2.04 (1.59, 2.62)
.
Overall (I-squared = 73.4%, p = 0.001) 1.40 (0.94, 2.08)
NOTE: Weights are from random effects analysis
1
Odds ratio of low parental education

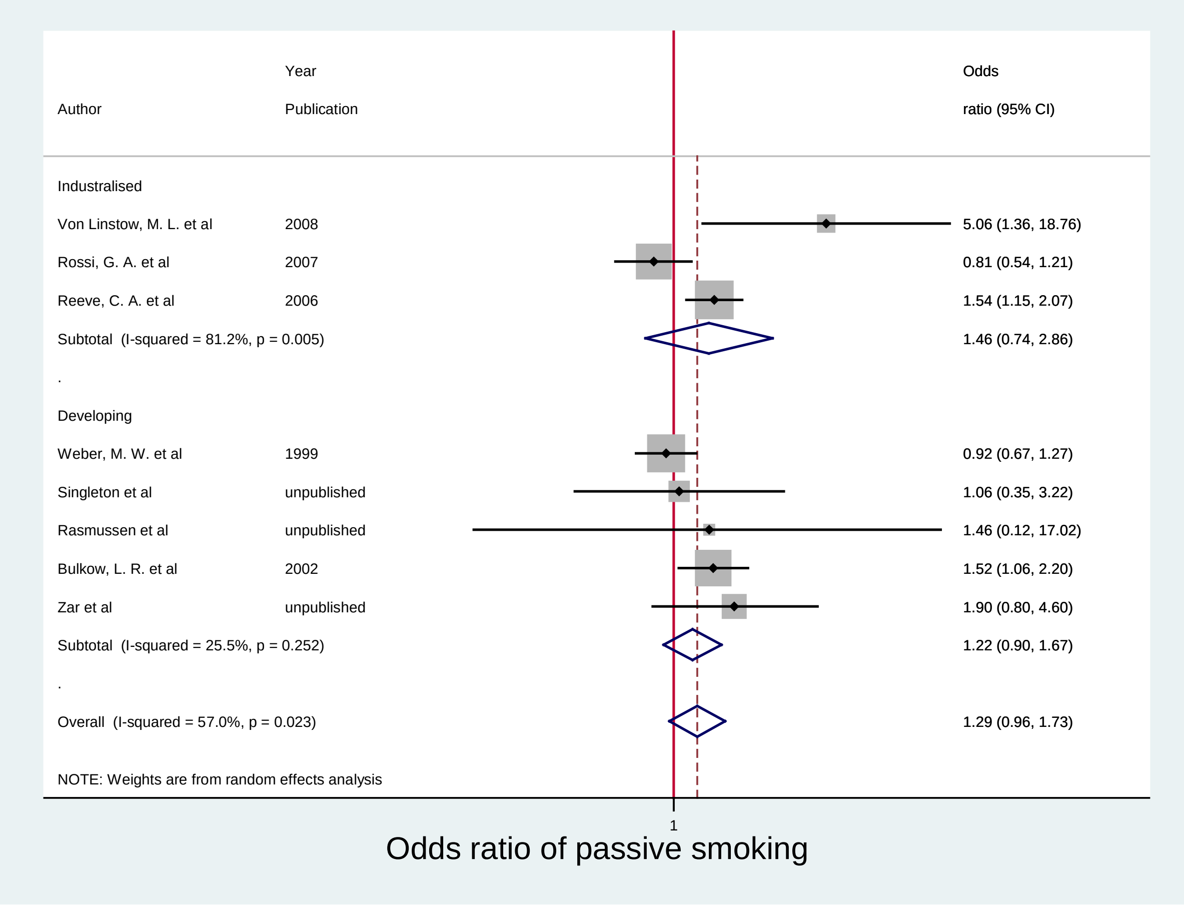
Author
Year
Publication
Odds
ratio (95% CI)
Industrialised
Von Linstow, M. L. et al 2008 5.06 (1.36, 18.76)
Rossi, G. A. et al 2007 0.81 (0.54, 1.21)
Reeve, C. A. et al 2006 1.54 (1.15, 2.07)
Subtotal (I-squared = 81.2%, p = 0.005) 1.46 (0.74, 2.86)
.
Developing
Weber, M. W. et al 1999 0.92 (0.67, 1.27)
Singleton et al unpublished 1.06 (0.35, 3.22)
Rasmussen et al unpublished 1.46 (0.12, 17.02)
Bulkow, L. R. et al 2002 1.52 (1.06, 2.20)
Zar et al unpublished 1.90 (0.80, 4.60)
Subtotal (I-squared = 25.5%, p = 0.252) 1.22 (0.90, 1.67)
.
Overall (I-squared = 57.0%, p = 0.023) 1.29 (0.96, 1.73)
NOTE: Weights are from random effects analysis
1
Odds ratio of passive smoking

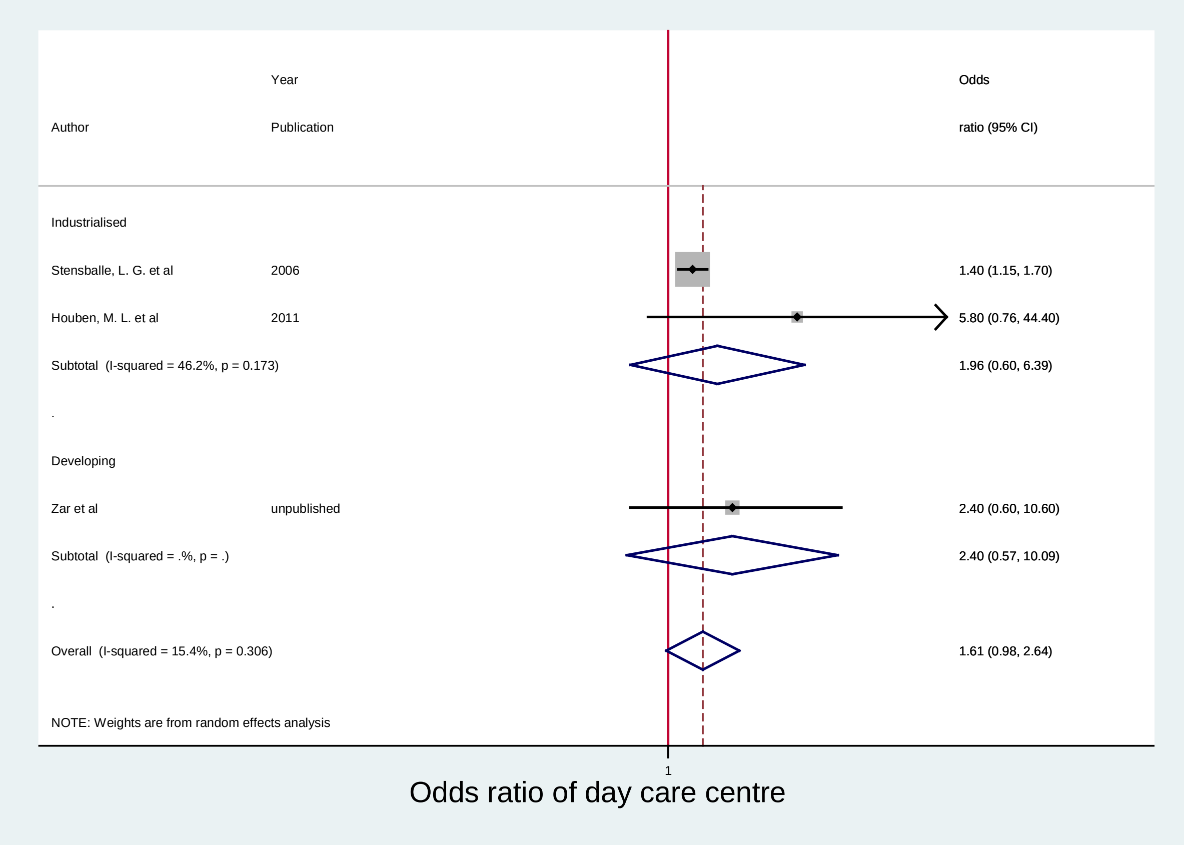
Author
Year
Publication
Odds
ratio (95% CI)
Industrialised
Stensballe, L. G. et al
2006
1.40 (1.15, 1.70)
Houben, M. L. et al
2011
5.80 (0.76, 44.40)
Subtotal (I-squared = 46.2%, p = 0.173)
1.96 (0.60, 6.39)
.
Developing
Zar et al
unpublished
2.40 (0.60, 10.60)
Subtotal (I-squared = .%, p = .)
2.40 (0.57, 10.09)
.
Overall (I-squared = 15.4%, p = 0.306)
1.61 (0.98, 2.64)
NOTE: Weights are from random effects analysis
1
Odds ratio of day care centre

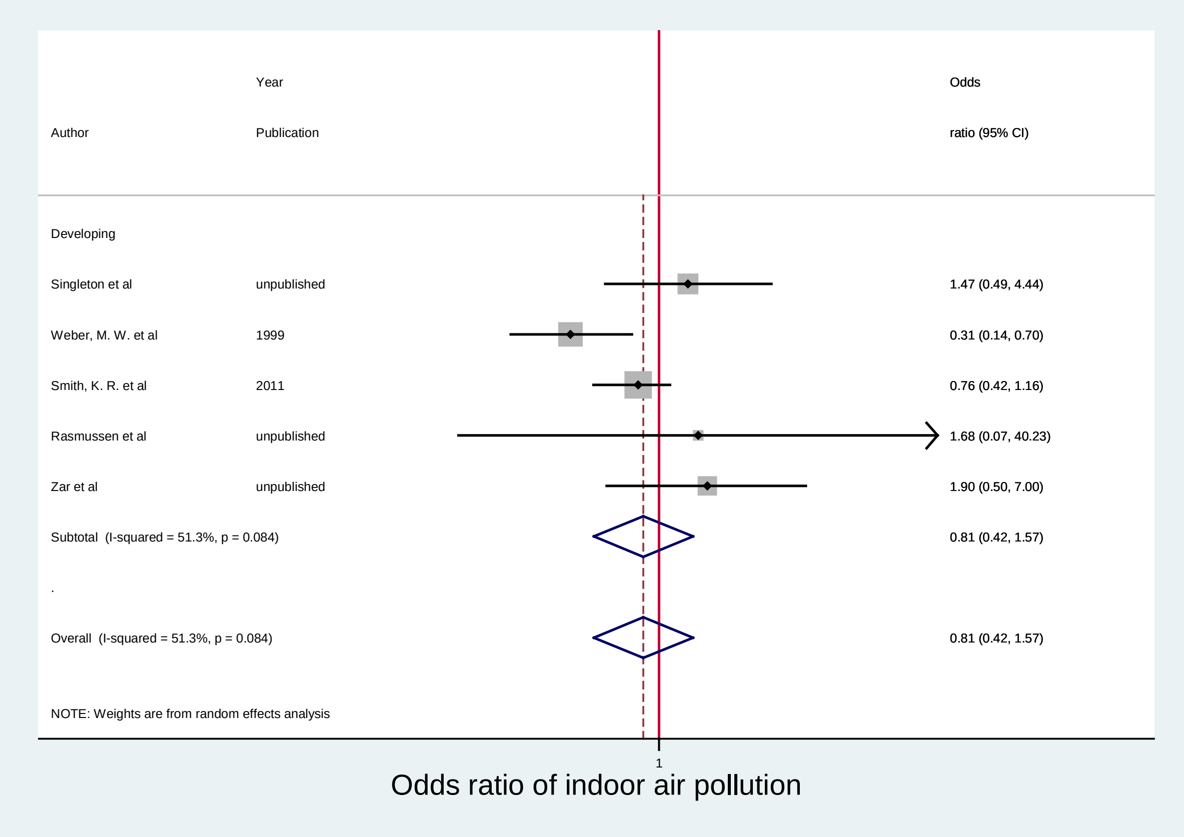
Author
Year
Publication
Odds
ratio (95% CI)
Developing
Singleton et al
unpublished
1.47 (0.49, 4.44)
Weber, M. W. et al
1999
0.31 (0.14, 0.70)
Smith, K. R. et al
2011
0.76 (0.42, 1.16)
Rasmussen et al
unpublished
1.68 (0.07, 40.23)
Zar et al
unpublished
1.90 (0.50, 7.00)
Subtotal (I-squared = 51.3%, p = 0.084)
0.81 (0.42, 1.57)
.
Overall (I-squared = 51.3%, p = 0.084)
0.81 (0.42, 1.57)
NOTE: Weights are from random effects analysis
1
Odds ratio of indoor air pollution

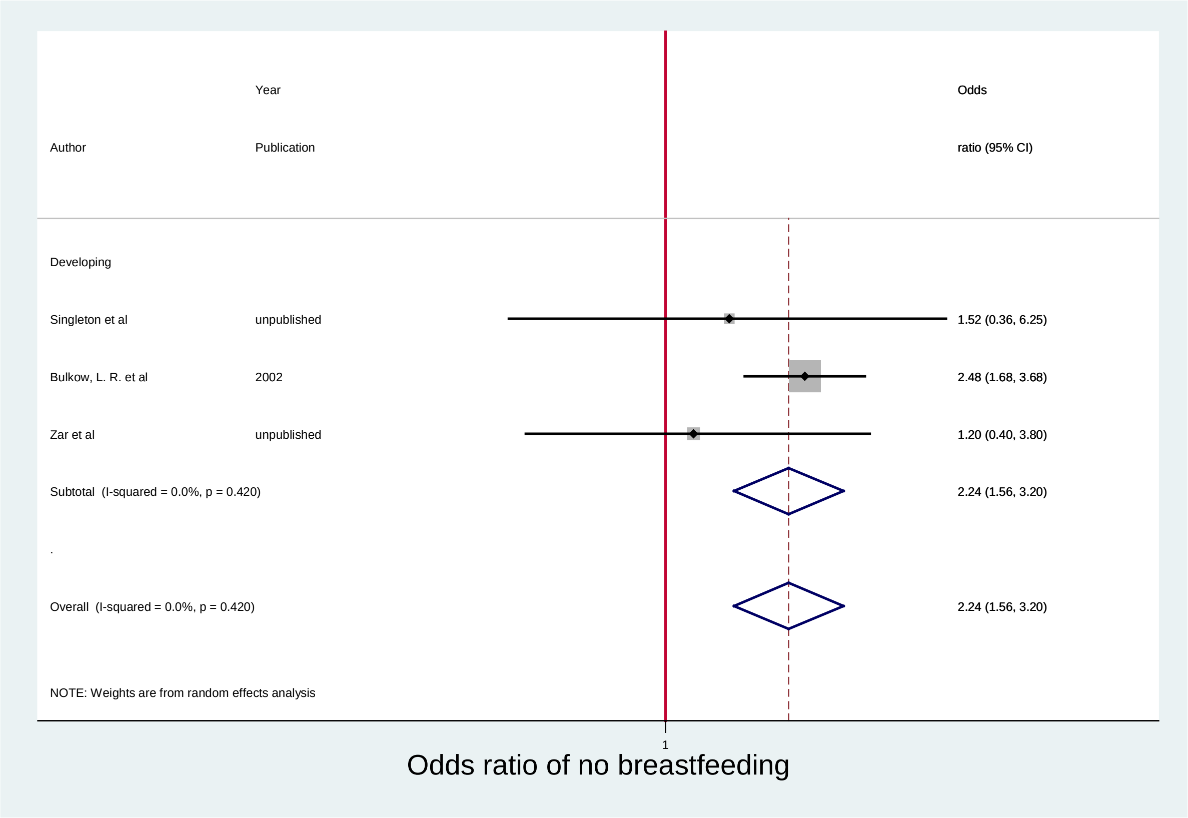
Author
Year
Publication
Odds
ratio (95% CI)
Developing
Singleton et al
unpublished
1.52 (0.36, 6.25)
Bulkow, L. R. et al
2002
2.48 (1.68, 3.68)
Zar et al
unpublished
1.20 (0.40, 3.80)
Subtotal (I-squared = 0.0%, p = 0.420)
2.24 (1.56, 3.20)
.
Overall (I-squared = 0.0%, p = 0.420)
2.24 (1.56, 3.20)
NOTE: Weights are from random effects analysis
1
Odds ratio of no breastfeeding

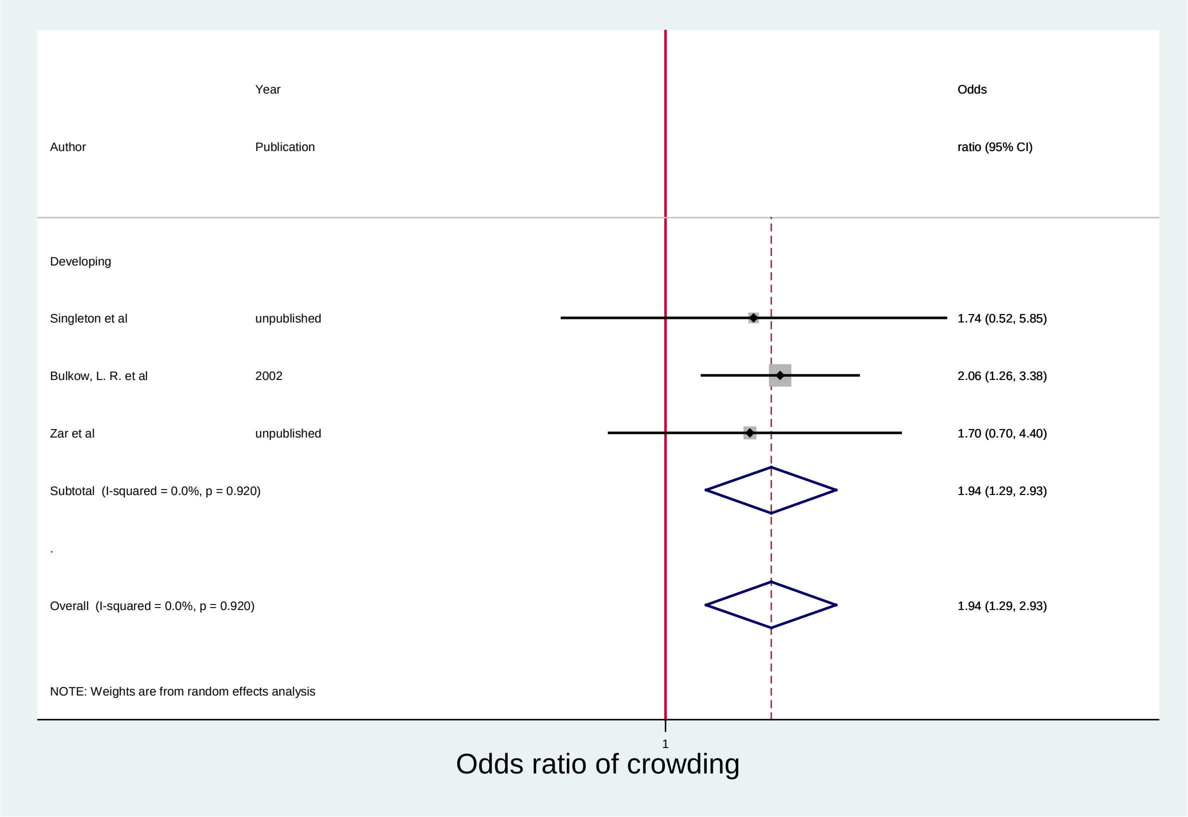
Author
Year
Publication
Odds
ratio (95% CI)
Developing
Singleton et al
unpublished
1.74 (0.52, 5.85)
Bulkow, L. R. et al
2002
2.06 (1.26, 3.38)
Zar et al
unpublished
1.70 (0.70, 4.40)
Subtotal (I-squared = 0.0%, p = 0.920)
1.94 (1.29, 2.93)
.
Overall (I-squared = 0.0%, p = 0.920)
1.94 (1.29, 2.93)
NOTE: Weights are from random effects analysis
1
Odds ratio of crowding

Appendix 7: Meta-estimate of odds ratio for risk factors including data from all studies (irrespective of quality scores)

Risk factor	Multivariable analysis		Multivariable and univariable analysis	
	No. of studies	Meta-estimate OR (95% CI)	No. of studies	Meta-estimate OR (95% CI)
**Prematurity (gestational age <37 weeks)**	2	-	9	1.47 (0.98-2.21)
**Low birth weight**	2	-	6	1.37 (0.85-2.21)
**Being male**	6	1.32 (1.24-1.40)	13	1.21 (1.12-1.32)
**Siblings**	6	1.53 (1.20-1.95)	12	1.62 (1.34-1.95)
**Maternal smoking**	4	1.34 (1.26-1.42)	7	1.36 (1.24-1.50)
**History of atopy**	1	-	5	1.47 (1.16-1.87)
**Low parental education**	4	1.23 (0.73-2.09)	7	1.77 (0.91-3.46)
**Passive smoking**	4	1.40 (0.65-3.00)	9	1.23 (0.95-1.60)
**Daycare centre attendance**	2	-	3	1.61 (0.98-2.64)
**Indoor air pollution**	4	0.69 (0.35-1.37)	6	0.86 (0.57-1.31)
**No breastfeeding**	1	-	3	2.24 (1.56-3.20)
**Crowding (>7 persons in household)**	1	-	3	1.94 (1.29-2.93)
**Multiple births**	1	-	3	1.41 (0.98-2.03)
**HIV**	2	-	3	3.74 (2.47-5.66)
**Lack of plumbed water**	2	-	3	1.46 (0.76-2.79)

Appendix 8: Sensitivity analysis of including "low quality" studies (with quality score ≤6.25)

Risk factor	Final estimate		Sensitivity analysis (including "low-quality" studies)	
	No. of studies	Meta-estimate OR (95% CI)	No. of studies	Meta-estimate OR (95% CI)
**Prematurity (gestational age <37 weeks)**	7	1.96 (1.44-2.67)	9	1.47 (0.98-2.21)
**Low birth weight**	5	1.91 (1.45-2.53)	6	1.37 (0.85-2.21)
**Being male**	12	1.23 (1.13-1.33)	13	1.21 (1.12-1.32)
**Siblings**	11	1.60 (1.32-1.95)	12	1.62 (1.34-1.95)
**Maternal smoking**	7	1.36 (1.24-1.50)	7	1.36 (1.24-1.50)
**History of atopy**	5	1.47 (1.16-1.87)	5	1.47 (1.16-1.87)
**Low parental education**	6	1.40 (0.94-2.08)	7	1.77 (0.91-3.46)
**Passive smoking**	8	1.29 (0.96-1.73)	9	1.23 (0.95-1.60)
**Daycare centre attendance**	3	1.61 (0.98-2.64)	3	1.61 (0.98-2.64)
**Indoor air pollution**	5	0.81 (0.42-1.57)	6	0.86 (0.57-1.31)
**No breastfeeding**	3	2.24 (1.56-3.20)	3	2.24 (1.56-3.20)
**Crowding (>7 persons in household)**	3	1.94 (1.29-2.93)	3	1.94 (1.29-2.93)
**Multiple births**	2	-	3	1.41 (0.98-2.03)
**HIV**	1	-	3	3.74 (2.47-5.66)
**Lack of plumbed water**	1	-	3	1.46 (0.76-2.79)

Appendix 9: Comparison of risk factors for RSV associated ALRI and severe ALRI

Risk factor	RSV associated ALRI		Severe ALRI (aetiology not specified)	
	No. of studies	Meta-estimate OR (95% CI)	No. of studies	Meta-estimate OR (95% CI)
**Prematurity (gestational age <37 weeks)**	7	1.96 (1.44-2.67)	5	1.9 (1.3-2.8)
**Low birth weight**	5	1.91 (1.45-2.53)	4	3.2 (1.0-9.9)
**Being male**	12	1.23 (1.13-1.33)	6	1.5 (1.0-2.3)
**Siblings**	11	1.60 (1.32-1.95)	0	-
**Maternal smoking**	7	1.36 (1.24-1.50)	3	2.7 (1.0-7.8)
**History of atopy**	5	1.47 (1.16-1.87)	0	-
**Low parental education**	6	1.40 (0.94-2.08)	6	1.6 (1.0-2.6)
**Passive smoking**	8	1.29 (0.96-1.73)	4	2.4 (1.0-5.8)
**Daycare centre attendance**	3	1.61 (0.98-2.64)	3	3.7 (0.7-20.2)
**Indoor air pollution**	5	0.81 (0.42-1.57)	5	1.6 (1.1-2.3)
**Crowding (>7 persons in household)**	3	1.94 (1.29-2.93)	3	1.9 (1.5-2.5)
**No breastfeeding**	3	2.24 (1.56-3.20)	6	2.3 (1.4-3.9)
**Lack of exclusive breastfeeding**	2	-	10	2.3 (1.4-3.9)
**Multiple births**	2	-	0	-
**HIV**	1	-	2	4.6 (2.2-9.7)
**Crowding (≥2 persons/room in household)**	2	-	4	2.2 (1.8-2.7)
**Malnutrition**	1	-	10	3.2 (1.6-6.4)
**Previous illness**	2	-	5	1.7 (0.8-3.4)
**Incomplete immunization**	0	-	5	1.8 (1.3-2.5)
**Vitamin D deficiency**	0	-	3	7.3 (2.5-21.5)
**Anemia**	0	-	5	3.9 (2.4-6.3)
**Zinc deficiency**	0	-	2	0.5 (0.3-0.9)
**Birth interval**	0	-	2	1.4 (0.9-2.2)
**Birth order**	0	-	3	2.1 (1.2-4.4)
**Vitamin A deficiency**	0	-	1	-
**High altitude**	1	-	0	-
**Lack of plumbed water**	1	-	0	-
